# pH-Responsive Epoxy Coating Incorporating a Novel Schiff Base-Loaded UiO-66-NH2 with an Encapsulated Shell for Long-Term Steel Corrosion Protection

**DOI:** 10.1021/acsomega.5c06277

**Published:** 2025-11-26

**Authors:** Tahere Miri, Davod Seifzadeh, Yunus Kara, Burak Dikici, Ozlem Gundogdu, Sertan Aytaç, Hadi Basharnavaz

**Affiliations:** † Department of Chemistry, 185149 University of Mohaghegh Ardabili, Ardabil 5619911367, Iran; ‡ Department of Chemistry, Sciences Faculty, 37503 Atatürk University, Erzurum 25240, Turkey; § Department of Mechanical Engineering, Ataturk University, Erzurum 25240, Turkey; ∥ Kaman Vocational School, Department of Food Technology, Ahi Evran University, Kırsehir 40100, Turkey; ⊥ Department of Chemistry, College of Science, 48537 Yazd University, Yazd 89195-741, Iran

## Abstract

To address the low corrosion resistance of conventional epoxy coatings, a smart nanocomposite was designed by using UiO-66-NH2, a metal–organic framework (MOF), loaded with a new Schiff base as an inhibitor. The MOFs were encapsulated with a pH-responsive shell, allowing for the controlled release of the inhibitor. The crystallinity of UiO-66-NH2 remained preserved after encapsulation, and spherical particles were observed with an apparent size increase following encapsulation. The specific surface area of UiO-66-NH2 decreased from 860.18 to 131.06 m^2^ g^–1^ and pore volume from 1.9745 to 0.2871 cm^3^ g^–1^, indicating efficient pore occupation by the inhibitor. This change resulted in a loading efficiency of 30.5% and an encapsulation efficiency of 43.9%, which contribute to the enhanced corrosion resistance of the coating. The encapsulated UiO-66-NH2 exhibited smart behavior, releasing the highest amount of inhibitor at pH 12. The addition of UiO-66-NH2 and its encapsulated form eliminated micrometric defects in the coating, and the MOFs were uniformly distributed throughout the epoxy matrix. The incorporation of UiO-66-NH2 reduced the surface roughness of the epoxy coating from 3.909 to 3.260 μm, with a further decrease to 1.851 μm upon addition of its encapsulated form. Electrochemical impedance spectroscopy (EIS) studies in 0.2 M HCl revealed enhanced corrosion resistance after UiO-66-NH2 addition, mainly due to MOF-coating interactions, eliminating structural pores. After 6 weeks of immersion, the epoxy coating with encapsulated UiO-66-NH2 achieved a polarization resistance (*R*_p_) of 327.33 MΩ cm^2^, surpassing the neat epoxy coating by ∼41 times and the UiO-66-NH2-containing coating by ∼10 times. Potentiodynamic polarization (PDP) and postcorrosion morphology aligned well with EIS results. Density functional theory calculations confirmed the potential for chemical and physical adsorption of Schiff base molecules onto the steel. The coating designed in this study shows potential for protecting steel in industrial environments, including chemical processing plants and water treatment facilities.

## Graphical abstract

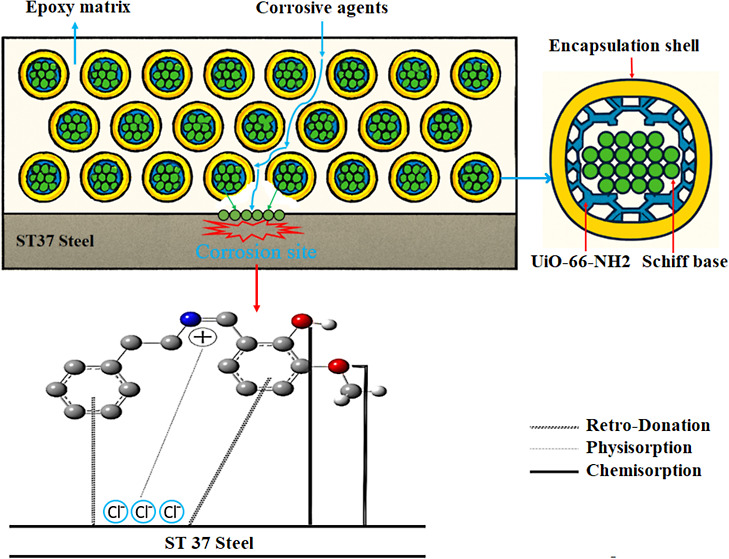

## 1 Introduction

Corrosion protection of steel has been widely studied due to steel’s fundamental applications in many industrial sectors. Among the various methods of corrosion protection, such as cathodic protection, anodic protection, and the addition of corrosion inhibitors, coating is generally recognized as a highly efficient and versatile approach. Anticorrosion coatings include organic, inorganic, and metallic types. Organic coatings are essential due to their considerable thickness and superior corrosion resistance. Widely applied for steel protection, epoxy coatings are a key category of organic anticorrosion barriers. These coatings are known for their strong adhesion to metallic surfaces and their high chemical stability. They also show good barrier properties against corrosive agents. However, similar to many other anticorrosion coatings, these coatings possess a degree of porosity. The extent of these pores, which can vary depending on the type of epoxy coating, application method, and thickness, negatively affects the corrosion protection. Especially if the aqueous corrosive agents pass through the coating and reach the metal surface, the pH changes and gas production (due to corrosion) can lead to swelling and eventually tearing of the coating.^1^

Various methods, such as blending, chemical modification, and the addition of nanostructures, have been proposed to reduce the permeability of epoxy coatings to corrosive agents. Addition of nanomaterials is notable among the aforementioned approaches due to its simplicity, range of applicable nanomaterials, and beneficial properties of the resulting nanocomposites. Although adding corrosion inhibitors is not considered a method for reducing the permeability of epoxy coatings, it does lead to a significant increase in corrosion resistance. The corrosion inhibitor comes into play when the corrosive solution has penetrated the epoxy coating and reached the surface of the metal substrate. The direct addition of corrosion inhibitors is not a successful strategy for improving corrosion resistance because there is always the risk of their dissolution and gradual leaching from the coating, as well as unwanted interactions with the coating matrix. Therefore, researchers have proposed using nanostructures and corrosion inhibitors simultaneously as an established strategy to improve the anticorrosion features of coatings. In this approach, the corrosion inhibitor is usually either loaded inside the porous nanostructures or chemically grafted onto them. Then, the inhibitor-containing nanostructure, also called a nanocarrier, is incorporated into the coating in a suitable amount. Although the presence of the nanostructure reduces the permeability of the anticorrosion coating by mechanisms such as filling the pores and creating tortuous paths for the corrosive electrolyte, some electrolytes may still penetrate the coating over extended exposure periods. At this time, the corrosion inhibitor within or on the surface of the nanocarrier becomes active and prevents corrosion. The important issue is the mechanism of release of the corrosion inhibitor from the nanocarrier. Corrosion inhibitors are typically confined within nanocarriers by using pH-sensitive encapsulation. During corrosion, pH changes trigger the sheath to open, allowing the inhibitor to be released and function effectively. When the grafting method is used to immobilize the corrosion inhibitor on the nanocarrier, the chemical bond between the inhibitor and the nanocarrier is usually designed to be pH-sensitive, which provides the basis for the release of the inhibitor when corrosion occurs. Thus, the nanocarrier-containing coating releases the corrosion inhibitor only when it is needed. Therefore, there is no risk of gradual leaching of the corrosion inhibitor or unwanted chemical interactions with the coating matrix. Anticorrosion coatings that have such capabilities are generally called smart coatings.^2–4^

Several nanostructures have been used as nanocarriers to strengthen the corrosion resistance of epoxy coatings applied to steel.^5,6^ For instance, Zhang et al.^7^ used mesoporous silica nanocarriers to add 2,5-dimercapto-1,3,4-thiadiazole to an epoxy coating applied to carbon steel, which had a positive effect on corrosion resistance. They also reported that modifying mesoporous silica with triethoxysilane and poly­(acrylic acid) had a positive effect on the release capacity of the corrosion inhibitor. Jia and Zhang^8^ have introduced an environmentally friendly approach to synthesize reduced graphene oxide (rGO) and then used it for the development of a ternary nanocomposite consisting of rGO, SiO_2_ nanoparticles, and benzotriazole (BTA). This BTA@SN-rGO nanocomposite, noted for its high inhibitor loading capacity, is integrated inside an epoxy latex matrix to develop a smart corrosion-resistant coating. Farzi et al.^9^ encapsulated cerium nitrate within poly­(urea-formaldehyde) microcapsules to develop a self-healing epoxy coating for carbon steel. The self-healing performance of the prepared coating was evaluated in a 0.6 M NaCl solution by using electrochemical impedance spectroscopy (EIS). Results showed that when the coating was compromised, cerium nitrate was released from the microcapsules, contributing to adequate corrosion protection through self-repair. In another study, Ubaid et al.^10^ tested the effect of TiO_2_ nanotubes loaded with dodecylamine (DDA) and epoxy monomers on the corrosion resistance and self-healing behavior of epoxy coating on carbon steel. The results indicated an improvement in corrosion resistance and the formation of self-healing properties in a 3.5 wt % NaCl solution due to the release of DDA and epoxy monomers, respectively. Also, the enhanced corrosion resistance of epoxy coatings containing 1 wt % sodium montmorillonite loaded with bis­(1-butyl-3-methylimidazolium) zinc tetrachloride ionic liquid compound is attributed to an ion-exchange mechanism between ionic liquid and sodium ions in the 3.5 wt % NaCl corrosive solution. This process facilitates the release and subsequent adsorption of ionic liquid onto the steel surface, thereby contributing to corrosion inhibition and supporting the coating’s self-healing behavior, as reported by Henriques et al.^11^

Metal–organic frameworks (MOFs), which are obtained by combining organic ligands with metal cations, are a group of one-, two-, or three-dimensional materials with a great variety in number and properties. Recognized as an important class of porous substances, they have attracted growing interest. Due to their high porosity, these compounds have a high capacity as nanocarriers for corrosion inhibitors in preparing smart coatings.^12–14^ Recently,^15^ a smart epoxy coating was prepared using a MOF compound called ZIF-8, which has shown good anticorrosion properties in a NaCl environment. For this purpose, Ti_3_C_2_ MXene 2D materials were first synthesized and modified by using in situ polymerization of dopamine. The modified MXene material was decorated with ZIF-8 as a corrosion inhibitor nanocarrier through subsequent coprecipitation and solvothermal procedures. Next, the obtained material (PM-Z) was loaded with two corrosion inhibitors (sodium phosphate and sodium glutamate) and added to the epoxy coating for smart anticorrosion action. Another investigation^16^ utilized ZIF-9-modified polyaniline-functionalized graphene oxide nanosheets to develop a pH-responsive epoxy coating for mild steel.

When corrosive environments with different pH values penetrate the coating or local changes in pH occur due to corrosion on the surface of the substrate metal, the chemical stability of the nanocarrier may be affected. For this reason, it is important to use MOF compounds with high chemical stability as nanocarriers. Zirconium-based metal–organic frameworks (Zr-MOFs) possess excellent thermal/chemical stability owing to the robust Zr–O bonds and the highly stable Zr_6_O_4_(OH)_4_ cluster core. These frameworks retain their structural integrity under harsh conditions, including elevated temperatures and exposure to acidic, basic, or saline environments. Their high surface area, three-dimensional porosity, and structural diversity make Zr-MOFs suitable for designing smart corrosion protection systems.^17,18^ Among the various zirconium-based MOFs, UiO-66, which forms through the coordination of terephthalic acid linkers with Zr^4+^ cations, has recently attracted considerable attention for its role in smart corrosion protection systems. This MOF compound has recently been used by Ramezanzadeh et al.^19^ as an inhibitor nanocarrier for the simultaneous loading of a green inhibitor and inorganic zinc cations in a smart epoxy coating on ST-12 steel. Additionally, Dabaleh et al.^18^ highlighted the potential of Zr-based MOFs to enhance the corrosion resistance of epoxy coatings through their integration with conductive polymers. Their study revealed that nanohybrids comprising polyaniline and varying proportions of UiO-66 form a core–shell architecture and facilitate pH-sensitive release of the inhibitor in a saline environment, thereby contributing to active corrosion protection.

Compared with UiO-66, UiO-66-NH2 offers distinctive advantages for the development of smart corrosion-protective epoxy coatings. The incorporation of amino functionalities enhances chemical interaction with the epoxy matrix, facilitating superior dispersion and stronger interfacial bonding through potential covalent linkages. Moreover, this modification not only retains the inherent thermal and chemical stability of UiO-66 but also imparts enhanced pH-responsive behavior, enabling a more efficient release of corrosion inhibitors under aggressive environmental conditions. In addition to their structural advantages, UiO-66-NH2 offers environmental benefits due to their nontoxic nature, chemical robustness, and minimal ecological footprint during synthesis. Therefore, UiO-66-NH2 was selected to formulate a high-performance smart epoxy nanocomposite for the application on ST-37 steel in this study. Also, a newly synthesized Schiff base compound, namely, 2-methoxy-6-((phenethylimino)­methyl), was loaded inside the UiO-66-NH2 pores. The use of a newly synthesized Schiff base corrosion inhibitor further contributes to sustainability by avoiding heavy metals and persistent inorganic compounds, often associated with long-term environmental risks. Additionally, the Schiff bases were selected as corrosion inhibitors due to their strong adsorption capacity onto the steel surface, facilitated by the azomethine (C=N) group. Especially in acidic environments, protonation enhances Schiff base solubility and enables both chemisorption and physisorption through coordination bonding and electrostatic interactions. The structural tunability, low toxicity, and minimal environmental impact of the Schiff bases further support their application in sustainable corrosion protection systems. Such choices reflect a conscious effort to develop smart anticorrosion coatings that align with green chemistry principles.

A set of analysis methods, including field emission scanning electron microscopy (FESEM), energy-dispersive X-ray spectrometry (EDX), atomic force microscopy (AFM), transmission electron microscopy (TEM), X-ray diffraction (XRD), Fourier transform infrared spectroscopy (FTIR), Brunauer–Emmett–Teller (BET), thermogravimetric analysis (TGA), water contact angle, and UV–vis spectrophotometry, will be used to characterize the UiO-66-NH2, its Schiff base-loaded form, and the epoxy coatings. The protective performance of the coatings against corrosion will be assessed through EIS analysis and potentiodynamic polarization (PDP), along with the morphological images after the corrosion tests. Density functional theory (DFT) is employed to analyze how the Schiff base interacts with the steel surface.

## 2 Experimental Details

### 2.1 Substrate

ST37 alloy discs, 5 cm in diameter and 0.5 cm in thickness, served as the metallic base material. First, copper wire was soldered to the back of the steel discs. Then, the samples were mounted with a polyester (Taba Co.). The metal surface, not connected to the copper wire, was polished using silicon carbide sandpapers with grit sizes ranging from 100 to 2000, until a 19.262 cm^2^ area was exposed from the polyester mount. Before the epoxy coatings were applied, the exposed metal surface was washed with water, degreased in ethanol, and dried. After the epoxy coatings were applied, the edges of the coated metal disc were completely sealed with two additional layers of epoxy. This sealing aimed to remove the risk of the corrosive test solution penetrating the interface between the steel disc and the polyester mount, potentially reaching the sides and back of the disk.

### 2.2 UiO-66-NH2

First, 0.686 mmol of zirconium chloride and 0.686 mmol of 2-aminoterephthalic acid were added to 40 mL of dimethylformamide (DMF) and stirred for 30 min. Next, the mixture was supplemented with 20 μL of deionized water and 1.2 mL of glacial acetic acid. Following autoclave treatment at 100 °C for 24 h, the resulting material was centrifuged and washed, first three times with DMF and then twice with ethanol. After 12 h of drying at 60 °C, UiO-66-NH2 particles were collected.^20^ Scheme 1 provides a schematic representation of the synthesis procedure for UiO-66-NH2.

**Scheme 1:**
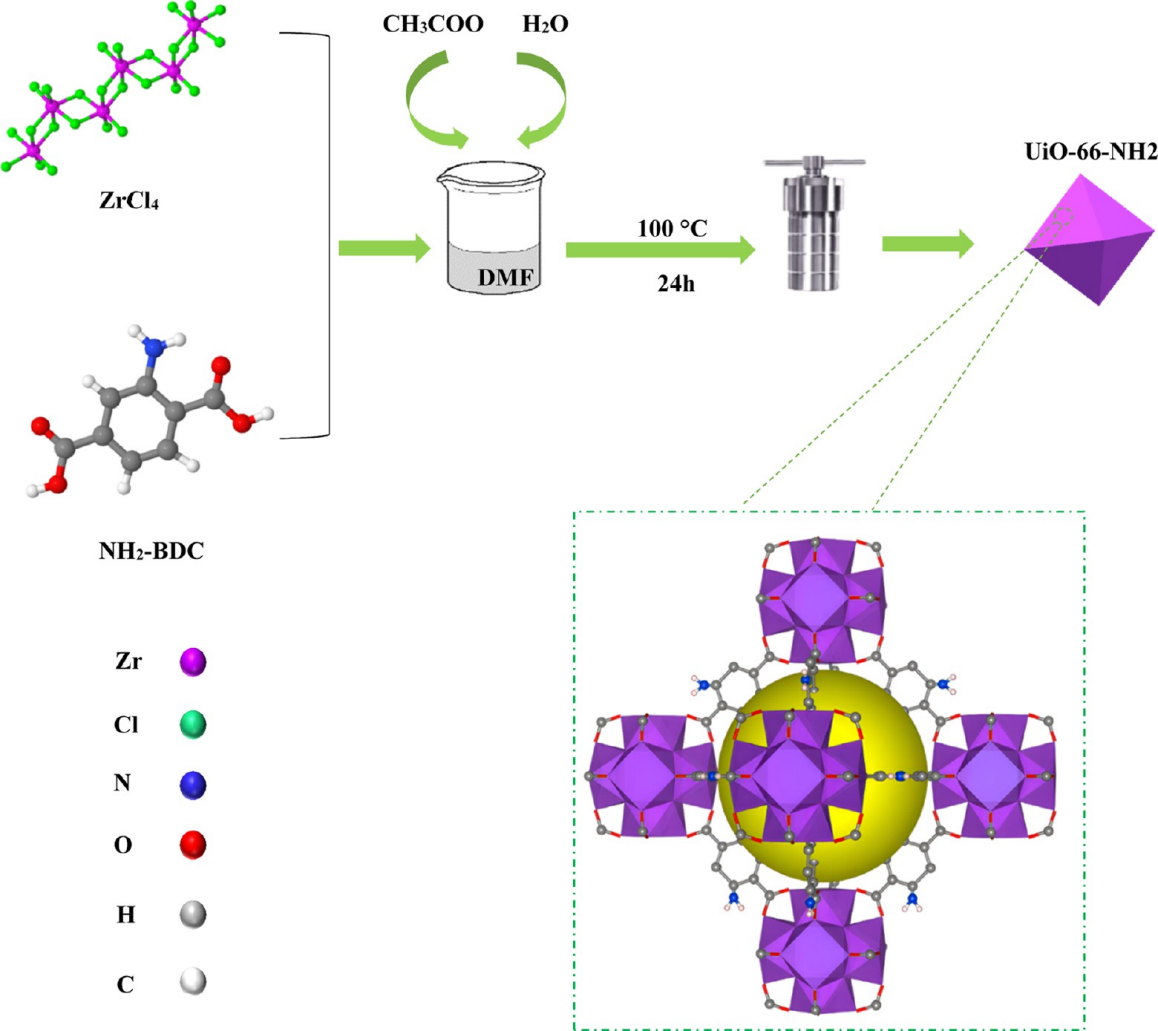
Schematic Illustrating the Synthesis Pathway of UiO-66-NH2

### 2.3 Schiff Base

The Schiff base was synthesized according to the procedures described in the literature.^21,22^ The 2-phenylethan-1-amine (1) (1 mmol) and 2-hydroxy-3-methoxybenzaldehyde (2) (1 mmol) were placed in the reaction vessel and directly exposed to microwave radiation (230 V/50 Hz, 700 W) for 5 min. Then, the desired Schiff base compound (3) (2-methoxy-6-((phenethylimino)­methyl)­phenol) was obtained in a short time and at a high yield (Scheme 2). No solvent or catalyst was used in the reaction. Reaction progress was tracked using thin layer chromatography under UV light (254 nm).

**Scheme 2:**
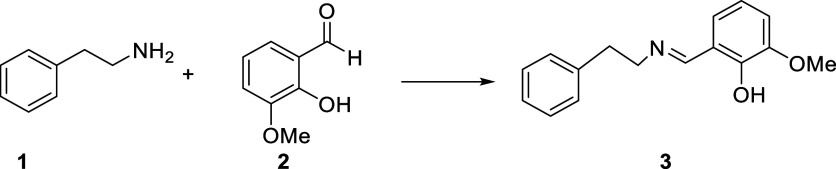
Synthesis of 2-Methoxy-6-((phenethylimino)­methyl)­phenol (3) ^1^H NMR (400 MHz, CDCl_3_): δ 13.88 (s, 1H, OH), 8.16 (s, 1H), 7.35–7.14 (m, 5H), 6.89 (d, *J* = 6.6 Hz, 1H), 6.83–6.70 (m, 2H), 3.89 (s, 3H), 3.83 (t, *J* = 5.1 Hz, 2H), 2.99 (t, *J* = 6.9 Hz, 2H). ^13^C NMR (100 MHz, CDCl_3_): δ 165.22, 151.99, 148.47, 139.16, 128.95, 128.54, 126.39, 122.85, 118.46, 117.78, 113.72, 60.67, 56.06, 37.36. FT-IR (cm^–1^): 2914, 1608 C=N, 1569, 1520, 1442 (C–N), 1339, 1315, 826. HRMS (ESI): [M + H]^+^ C_14_H_19_N_4_O; founded, 259.1584; calcd 259.1559

### 2.4 Loading of the Schiff Base

First, 0.08 g of UiO-66-NH2 was mixed with an ethanolic solution of the Schiff base (0.08 g in 10 mL). For better dispersion, the mixture was subjected to ultrasonic waves for 10 min. Then, it was stirred at room temperature for 24 h. A vacuum system was applied to the solution in a glass flask to ensure the evacuation of air from the pores of UiO-66-NH2. After air bubbles were removed from the pores, the flask was sealed for 30 min. As the air bubbles exited, the Schiff base molecules entered the pores. The procedure was carried out three times to achieve complete infiltration of Schiff base molecules into the internal pores. The mixture underwent high-speed centrifugation at 3500 rpm, and the Schiff base-loaded MOFs were then vacuum-dried at 70 °C for an extended overnight period. Following the designated loading period, the concentration of the Schiff base remaining in the supernatant was subsequently determined via UV–vis spectroscopy. Then, the loading capacity (LC %) was calculated using the equation provided below^23^

1 $$Loading\;capacity\;\left(LC\%\right)=\frac{Weight\;of\;loaded\;inhibitor}{Total\;weight\;of\;nanocarrier+loaded\;inhibitor}\times 100$$

### 2.5 Encapsulation

To encapsulate the Schiff base-loaded MOFs, a layer-by-layer method was employed using two successive layers of tannic acid and chitosan.^24^ Initially, two distinct solutions were prepared: Solution A, an aqueous solution of tannic acid at a concentration of 1 g L^–1^, and Solution B, a 1 g L^–1^ solution of chitosan dissolved in 2% (by weight) acetic acid. The pH levels of Solutions A and B were set to 3.4 and 5, respectively. For encapsulation, 0.08 g of the Schiff base-loaded MOFs was added to 5 mL of Solution A and stirred for 20 min. The mixture was then centrifuged, and the material was collected and washed three times with a 0.1 M NaCl solution. Next, the product from the previous step was added to Solution B, centrifuged, and rinsed three times using 0.1 M NaCl. These steps were repeated to ensure that successive bilayers of tannic acid and chitosan were adsorbed onto the Schiff base-loaded MOFs. Finally, the encapsulated MOFs were dried at room temperature. Also, the encapsulation efficiency (EE %) was calculated via the following equation^23^

2 $$Encapsulation\;Efficiency\;\left(EE\%\right)=\frac{Weight\;of\;loaded\;inhibitor}{Total\;weight\;of\;feeding\;inhibitor}\times 100$$

### 2.6 Application of the Epoxy Coatings

To prepare a neat epoxy coating, epoxy resin (KUMHO Epoxy resin KER 828) and polyamine hardener (PC205t) were mixed in appropriate proportions and stirred for 10 min at 700 rpm. To dilute the mixture, thinner (Extra 2, Arko Co) was added and stirred for 10 min. The weight ratio of resin/hardener/thinner was 1:1:2. The coating solution was gently brushed onto the steel alloy, and the thickness of the coating was precisely controlled using a digital thickness gauge to achieve approximately 70 ± 1 μm. After being exposed to laboratory conditions for about 24 h, the coated samples underwent curing in a digital oven at 100 °C for 1 h. To avoid cracking, the temperature was carefully increased at a steady rate of 5 °C per minute.

The epoxy coating incorporating UiO-66-NH2 and its encapsulated form was formulated by blending these materials into the hardener at a defined weight ratio. The mixtures were then subjected to ultrasonic treatment for 30 min to ensure the dispersion of the MOFs. Subsequently, the required quantities of epoxy resin and thinner were introduced, and the mixtures were agitated for 10 min. The hardener/resin/thinner ratio was maintained as in the neat epoxy coating. The concentration of UiO-66-NH2 and its encapsulated form in the coatings was set at 0.5 wt % relative to the total weight of the epoxy resin and hardener mixture. Hereafter, the neat coating, coating incorporating MOFs, and coating with encapsulated MOFs will be referred to as EP, EP-MOF, and EP-EnMOF, respectively.

### 2.7 Characterization of UiO-66-NH2 and Schiff Base

To verify the molecular composition of the synthesized Schiff base compound, FTIR (VERTEX 70v FTIR Spectrometer) and NMR analyses were conducted. ^1^H NMR and ^13^C NMR spectra were obtained at 400 and 100 MHz, utilizing CDCl_3_ as the solvent (Bruker Varian NMR). HRMS analysis of the synthesized Schiff base was performed using the Waters LCT Premier mass spectrometer.

The FTIR spectra of UiO-66-NH2 and its encapsulated form were also recorded by using a PerkinElmer Spectrum Rx1 instrument. The morphology of the synthesized UiO-66-NH2 before and after the encapsulation was studied using FESEM (ZEISS Sigma 300). Before the analysis, a thin gold layer was deposited on the samples to enhance the conductivity. The electron accelerator operated at a voltage of 5 kV. Also, the atomic percentage of each constituent element of UiO-66-NH2 was determined by using the EDX technique.

The size and shape of the synthesized UiO-66-NH2 particles were investigated by using TEM. This study was conducted with a Hitachi HighTech HT7700 device, operating at a voltage of 120 kV.

Additionally, the diffraction patterns of the pristine and encapsulated MOFs were recorded by using a Rigaku MiniFlex X-ray diffractometer equipped with a Cu Kα radiation source (λ = 1.5406 Å), operating at a scan rate of 4° min^–1^.

The thermal stability of UiO-66-NH2 and its encapsulated form was assessed via TGA using a LINSEIS STA PT1000 instrument under nitrogen flow with a heating rate of 10 °C min^–1^ over a temperature range of 0–700 °C.

To investigate the pH-sensitive release of the Schiff base molecules from encapsulated UiO-66-NH2, a Thermo Fisher Scientific NanoDrop One C UV–vis spectrophotometer was used. To achieve this, the encapsulated UiO-66-NH2 (0.01 g) was suspended in 10 mL of deionized water and subjected to 10 min ultrasonic treatment. The pH was then modified to the target values of 2, 4, 6, 8, 10, and 12 by carefully introducing appropriate amounts of HCl and NaOH solutions. UV–vis spectra were recorded over the 200–800 nm range for each pH level at specified time intervals.

### 2.8 Characterization of the Coatings

The morphological features of the epoxy coatings, both before and after the addition of MOFs, were analyzed by using FESEM (ZEISS Sigma 300). The samples were coated with a thin layer of gold to enhance the electrical conductivity of the surface. The analyses were conducted under an accelerating voltage of 5 kV. Given the importance of proper dispersion of nanostructures in anticorrosion coatings, thin layers (∼50 nm) of epoxy coatings containing MOFs and encapsulated MOFs were prepared using ultramicrotomy (Leica EM UC7). These layers were then studied by TEM (EM10C-100 kV) at various magnifications. The topographical changes of the epoxy coating after the addition of the MOFs were also investigated using a CoreAFM system with a silicon nitride cantilever operated in noncontact mode. To accurately estimate the changes in the surface roughness (*S*_a_), an extensive analysis area of 2500 μm^2^ was selected.

Water contact angle measurements were conducted by using the Biolin Scientific Attension Theta Flex contact angle meter to assess the impact of incorporating MOFs and encapsulated MOFs on the wettability of the epoxy coating. The volume of the water drop used was 5 μL. Images were captured continuously for 10 s from the moment of droplet deposition. The contact angles on both the right and the left sides of the water drop were measured throughout this period. Subsequently, mean contact angle values over the 10 s interval were calculated, along with corresponding error margins.

Long-term corrosion tests were conducted in a 0.2 M hydrochloric acid solution. EIS and PDP tests were performed by using a μAUTOLAB Type III device. A 5 cm-diameter disk working electrode was used for the corrosion tests. As previously described in Section 2.1, the working electrodes were meticulously mounted and sealed to completely prevent any possibility of corrosive solution leakage to the sides or back of the metal disk. The EIS tests were performed in potentiostatic mode within the 100 kHz to 10 mHz frequency range by applying an alternating voltage of 20 mV around the open circuit potential of the working electrodes. The PDP tests were conducted with a potential scanning speed of 1 mV s^–1^, starting from −300 mV relative to the corrosion potential (*E*_corr_) and continuing to +300 mV relative to *E*_corr_. Data from EIS and PDP tests were analyzed using Zview2 and Nova 1.6 software, respectively. To ensure the reproducibility of the electrochemical test results, each test was conducted at least three times, and the average results were calculated.

### 2.9 DFT Analysis

Quantum chemical simulations were performed using DFT based on the Becke-3-Lee–Yang–Parr (B3LYP) method, coupled with a 6–311 G (d, p) basis set within the Gaussian g09 program, to explore the underlying mechanism of Schiff base compound adsorption onto the steel surface. Geometry optimization calculations of the Schiff base molecule were carried out in the solvated phase.

## 3 Results and Discussion

### 3.1 Characterization of the Schiff Base and MOFs

The successful synthesis of the Schiff base was confirmed by FT-IR, NMR, and HRMS analyses (Figure S1). The FTIR spectrum of UiO-66-NH2 (Figure 1) displays distinct bands at 1578 cm^–1^ and 1658 cm^–1^, indicating C=C stretching vibrations of the benzene ring and coordinated carboxylate moieties, respectively. The bands at 1388 cm^–1^ and 1259 cm^–1^ correspond to the C–N stretching vibrations of 2-aminoterephthalic acid. The presence of primary amine groups is revealed by the symmetric and asymmetric N–H stretching vibrations at ≈3370 cm^–1^ and ≈3460 cm^–1^, respectively. Lastly, the Zr–O vibrations are evident at 768 cm^–1^ and 663 cm^–1^ in the FTIR spectrum of the UiO-66-NH2.^24–27^ After encapsulation, the IR bands of the encapsulating materials were not observed in the FTIR spectra (Figure 1). This can be attributed to the limited thickness of the encapsulating layers and the relatively weak IR absorption of these materials compared to the MOF itself. Additionally, the absence of the Schiff base IR bands might result from the confinement of Schiff base molecules inside the pores of MOFs. Other potential factors include the low quantity of Schiff base in comparison to the MOF host material, as well as the proximity and spectral overlap of Schiff base and MOF bands.

**Figure 1:**
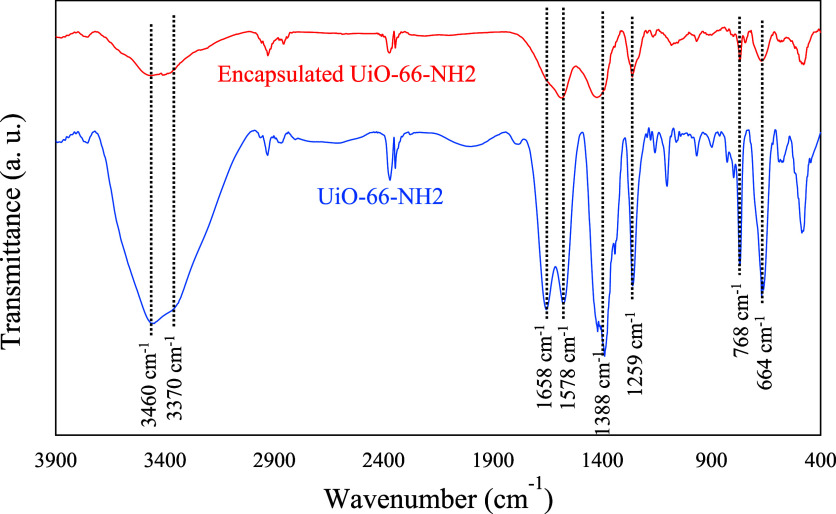
FTIR spectra of the UiO-66-NH2 and its encapsulated form.

The synthesized UiO-66-NH2 exhibited a spherical morphology that remained unchanged after encapsulation (Figure 2a,b). However, the particle size increased to some extent following Schiff base loading and encapsulation. The loading of the Schiff base inside the pores of MOFs may have expanded the particle’s overall dimensions by filling and potentially stretching the porous framework. Additionally, encapsulation with a polyelectrolyte shell formed an outer layer around the particles. These processes collectively contributed to the observed increase in the particle size. The atomic percentages of C, N, O, and Zr in UiO-66-NH2, as determined by EDX, were found to be 69.29%, 3.23%, 22.73%, and 4.75%, respectively (Figure 2c). While EDX has limitations in accurately quantifying light elements with atomic numbers below 11, such as C, N, and O, the detection of characteristic peaks for these elements, alongside Zr, confirms their presence in the structure of the synthesized material. The TEM image of UiO-66-NH2 indicates that the synthesized particles have a nearly uniform size distribution (Figure 2d).

**Figure 2:**
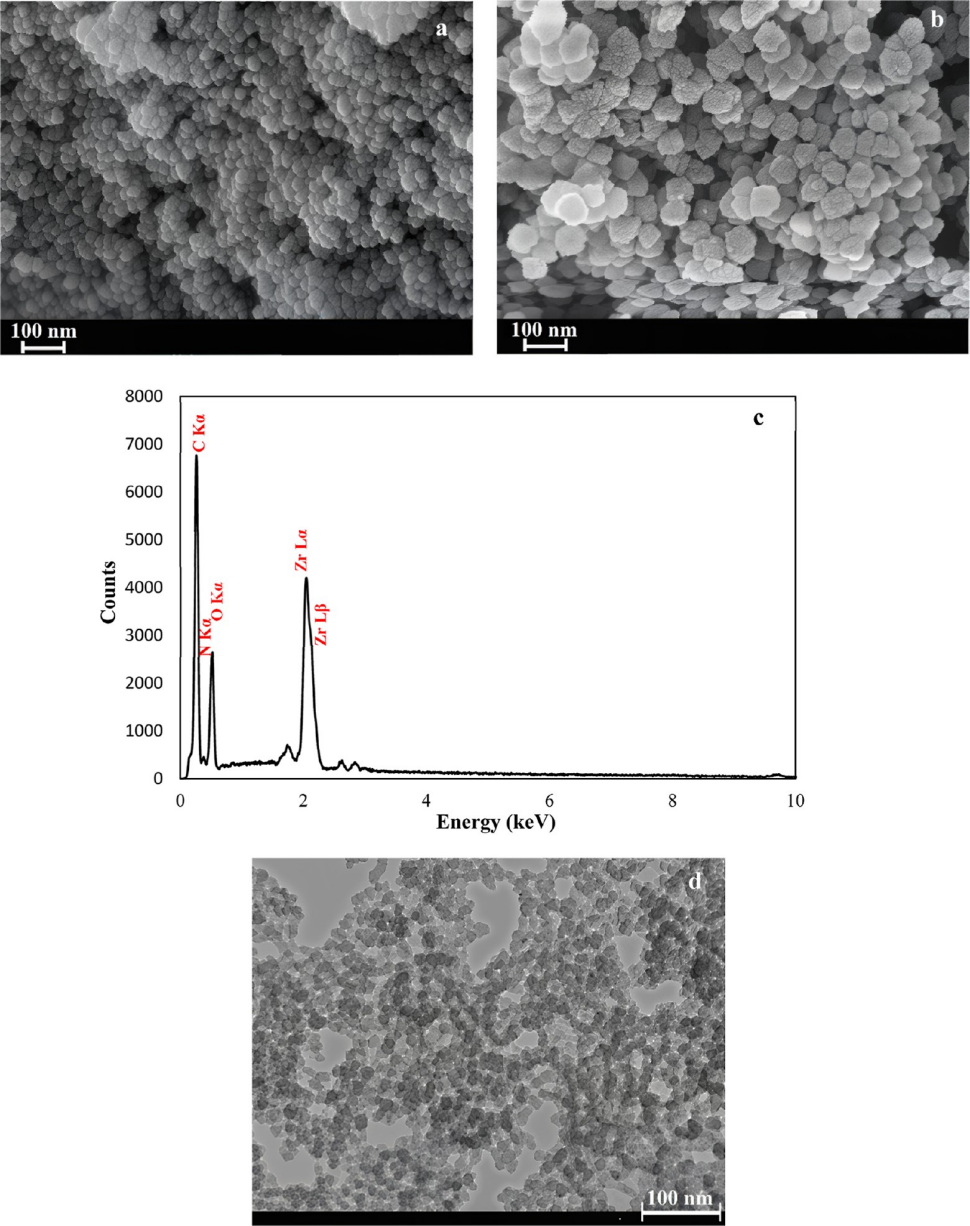
FESEM images of the UiO-66-NH2 before (a) and after (b) encapsulation, EDX spectrum of the UiO-66-NH2 (c), and TEM image of the UiO-66-NH2 (d).

The XRD peaks observed at 2θ values of 7.5°, 8.7°, 12.2°, 17.3°, 19.3°, 25.8°, and 30.8°, corresponding to *d*-spacings of 11.77, 10.15, 7.24, 5.12, 4.59, 3.45, and 2.90 Å, respectively, can be assigned to the (111), (200), (220), (400), (420), (600), and (711) crystalline planes (Figure 3). The XRD pattern agrees with the XRD pattern of UiO-66-NH2 and its simulated one (CCDC No. 889529).^28,29^ Comparable XRD patterns were recorded for UiO-66-NH2 and its encapsulated form, indicating that the MOF’s crystalline structure remained intact throughout the Schiff base loading and encapsulation processes, confirming its robust framework stability.

**Figure 3:**
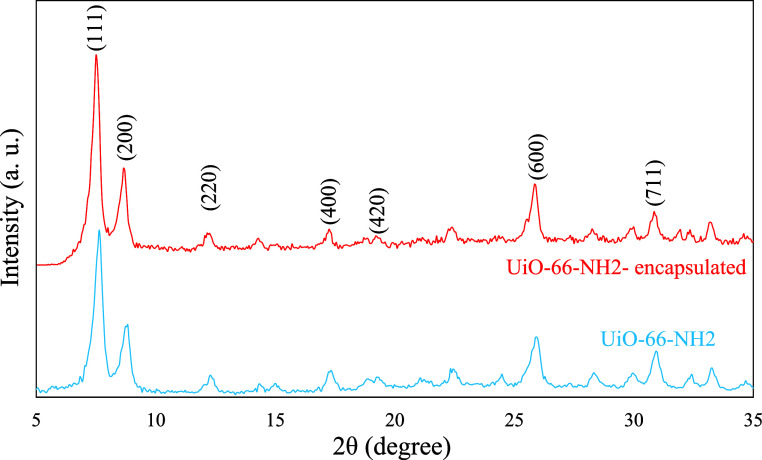
XRD patterns of the UiO-66-NH2 prior to and following encapsulation.

The TGA curve of UiO-66-NH2 (Figure 4) reveals a three-step weight loss profile indicative of its structural and thermal behavior. The initial ∼17% weight loss below 150 °C corresponds to the desorption of physically adsorbed water and residual solvent commonly retained in the porous framework. The second step, an ∼18% weight loss between 150 and 300 °C, is attributed to the removal of coordinated water and partial dehydroxylation of the Zr_6_ clusters. The final ∼20% weight loss from 300 to 600 °C signifies the progressive breakdown of the organic linkers (2-aminoterephthalic acid), ultimately leading to structural collapse.^30,31^ The encapsulated UiO-66-NH2 exhibited an enhanced thermal stability. The observed weight losses of ∼5% (0–150 °C), ∼13% (150–300 °C), and ∼37% (300–600 °C) indicated a delayed decomposition onset and attenuated weight loss in the early degradation stages. This behavior suggests that multilayer encapsulation by tannic acid and chitosan effectively retards solvent evaporation and linker decomposition, likely through hydrogen bonding and interfacial interactions that stabilize the hybrid structure under thermal stress.

**Figure 4:**
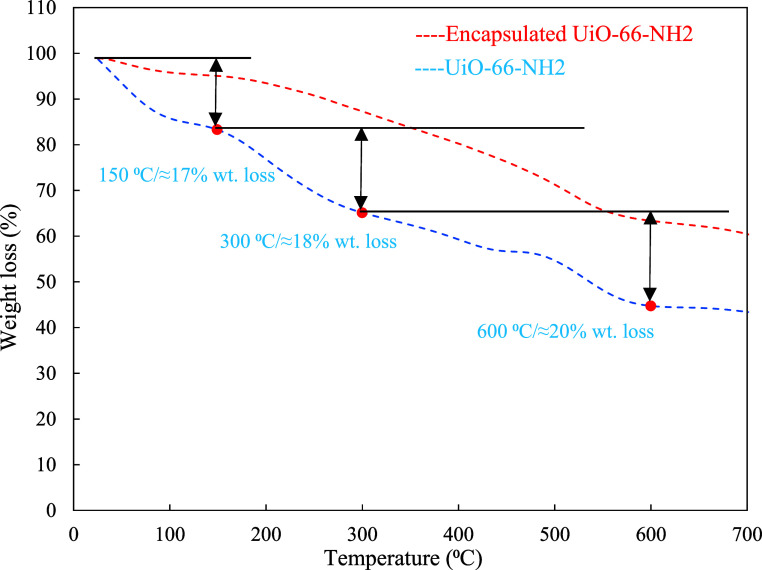
TGA curves of the UiO-66-NH2 and its encapsulated form.

Type adsorption–desorption isotherms of UiO-66-NH2 and its encapsulated form were recorded (Figure 5a,b, respectively). The quantitative results from the BET analyses are summarized in Table 1. The BET plot revealed significant changes in the properties of UiO-66-NH2 after Schiff base loading. The BET plot indicates a substantial reduction in various parameters, including *V*_m_ from 197.63 to 30.113 cm^3^ g^–1^, *a*_s,BET_ from 860.18 to 131.06 m^2^ g^–1^, BET constant from 3614.5 to 119.61, total pore volume from 1.9745 to 0.2871 cm^3^ g^–1^, and mean pore diameter from 9.1817 to 8.7629 nm. These results suggest a significant decrease in the surface area and pore volume of the MOFs, likely due to the filling of the internal pores by the Schiff base molecules, which restricts the available space for N_2_ gas adsorption. Additionally, the BJH plot (Figure 4c,d) parameters, including *V*_p_, *r*_p,peak_, and *a*_p_, show decreases from 1.6075 cm^3^ g^–1^, 33.24 nm, and 134.47 m^2^ g^–1^ to 0.2494 cm^3^ g^–1^, 33.24 nm, and 29.503 m^2^ g^–1^, respectively, except for the *r*_p_, peak, which remains unchanged at 33.24 nm. This further supports the conclusion that the Schiff base molecules effectively occupy the MOF’s pores, resulting in decreased porosity while maintaining the same peak pore radius. These findings indicate successful encapsulation of the Schiff base compound within the pores of the MOFs, significantly altering its surface and pore characteristics. The recorded pore diameters for UiO-66-NH2, prior to and following encapsulation, are 2 to 50 nm. Thus, the synthesized MOFs fall into the category of mesoporous materials according to an IUPAC definition.^32^ While the N_2_ adsorption–desorption isotherm exhibits type-I characteristics (typically associated with microporous structures), pore size analysis demonstrated a mesoporous structure. This contradiction may stem from synthesis-related features such as structural defects, missing linkers, or interparticle voids formed during crystallization. These factors can introduce secondary porosity even in frameworks originally designed to be microporous.

**Figure 5:**
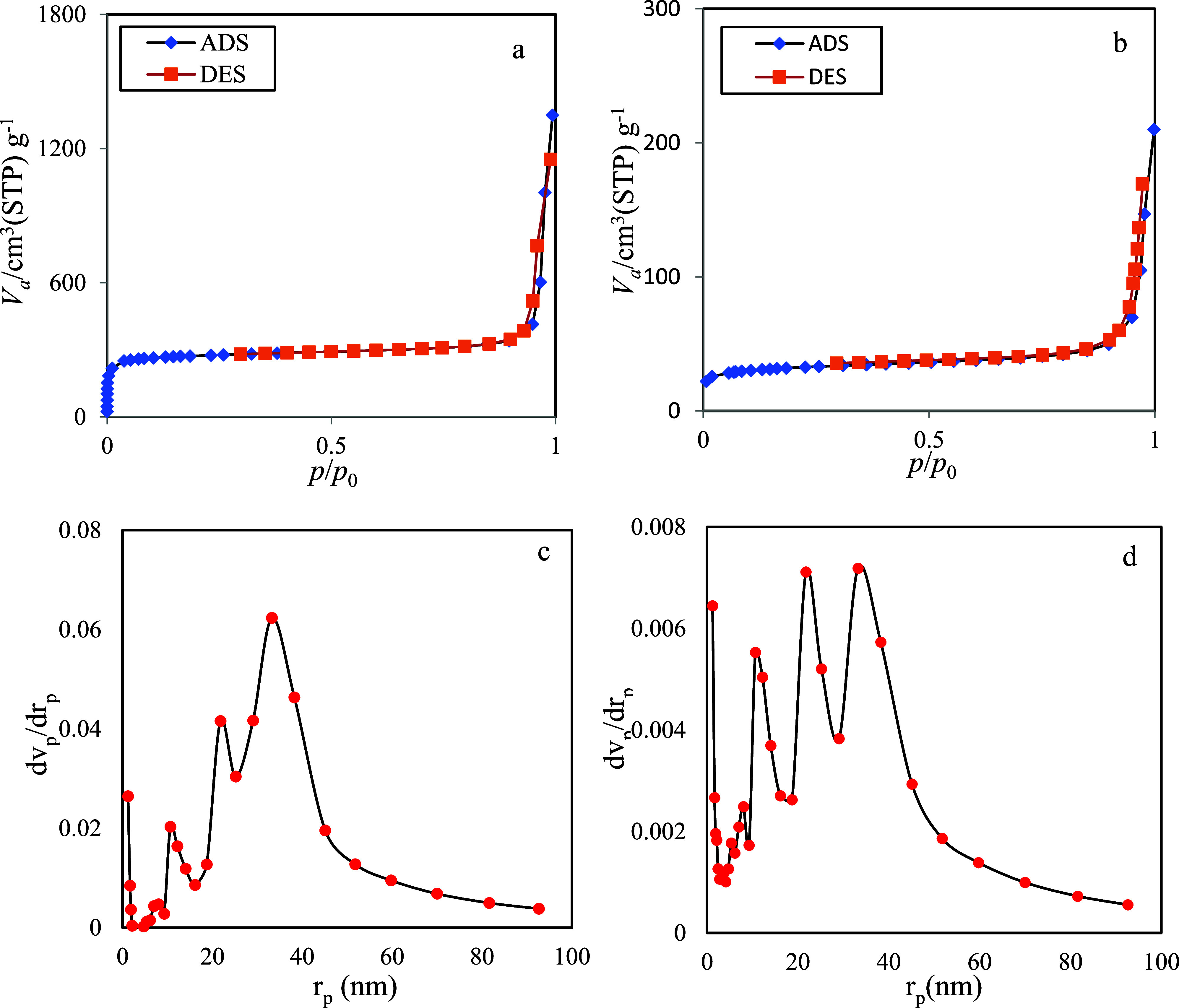
Adsorption/desorption and BJH plots of the UiO-66-NH2 (a, c) and its encapsulated form (b, d).

**Table 1:** BET and BJH Parameters of the UiO-66-NH2 and Its Encapsulated Form

BET plot		
sample	UiO-66-NH2	encapsulated UiO-66-NH2
*V*_m_ (cm^3^ g^–1^)	197.63	30.113
*a*_s, BET_ (m^2^ g^–1^)	860.18	131.06
C	3614.5	119.61
total pore volume (*p*/*p*_0_ = 0.990) (cm^3^ g^–1^)	1.9745	0.2871
mean pore diameter (nm)	9.1817	8.7629
BJH plot		
sample	UiO-66-NH2	encapsulated UiO-66-NH2
plot data	adsorption branch	adsorption branch
*V*_p_ (cm^3^ g^–1^)	1.6075	0.2494
*r*_p,peak_(Area) (nm)	33.24	33.24
*a*_p_ (m^2^. g^–1^)	134.47	29.503

The solubility of the synthesized Schiff base in 0.2 M HCl, which was used as the corrosion testing medium, was calculated to be 49 mg mL^–1^. The pH-responsive release of Schiff base molecules from the UiO-66-NH2 nanocarrier, triggered by corrosion-induced pH changes, was investigated by using UV–visible spectroscopy across various pH levels. The results indicated the highest release at pH 12, with significant release also observed at acidic pH values of 2 and 4 (Figure 6). These findings confirm that the inhibitor release occurs in a pH-dependent manner. A total of 35.12 mg of inhibitor was successfully loaded into the nanocarriers, as determined by UV–visible spectroscopy (Figure 5). Given that the initial inhibitor amount and the quantity of nanocarrier used were both 0.08 g, the loading capacity and encapsulation efficiency were calculated to be 30.5% and 43.9%, respectively, based on eqs 2 and 3. The calculated LC % and EE % values confirmed the successful integration of the Schiff base molecules into the pores of UiO-66-NH2. Also, these values correlate with the observed reductions in *V*_m_, *a*_s,BET_, *V*_p_, and *a*_p_ in BET and BJH analyses, indicating that a considerable fraction of the MOF’s internal porosity has been occupied by the encapsulated Schiff base molecules.

**Figure 6:**
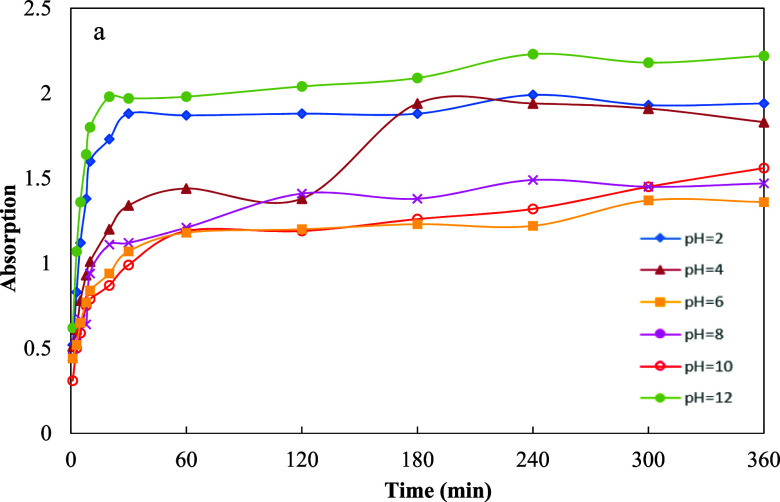
UV–visible spectra illustrating the release of Schiff base from the UiO-66-NH2 nanocarrier at varying pH levels.

### 3.2 Characterization of the Coatings

The morphological features of the applied epoxy coatings are illustrated in Figure 7. At first glance, the neat epoxy coating appears uniform; however, a closer examination reveals structural defects (Figure 7a). A defect is highlighted with greater clarity in Figure 7b. These defects may arise due to solvent evaporation during the heat treatment process. Furthermore, insufficient cross-linking in specific regions could result in structural weakness within the coating, ultimately leading to defect formation. These defects compromise the coating’s integrity by creating pathways for corrosive agents like water, oxygen, etc., to penetrate and reach the underlying steel. As a result, the protective barrier is weakened, accelerating localized corrosion and reducing the overall effectiveness of the coating in preventing steel corrosion.

**Figure 7:**
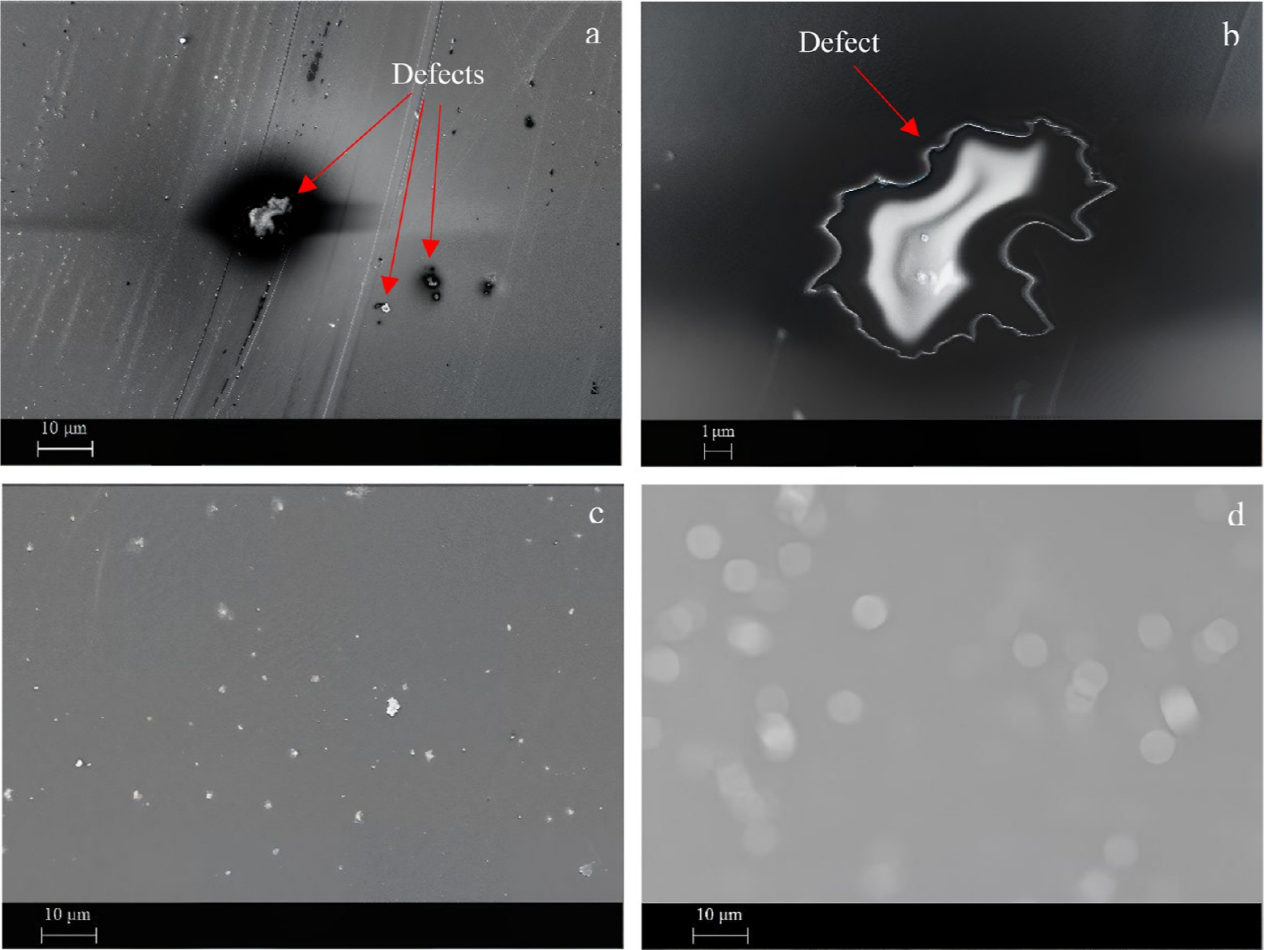
FESEM images of the EP (a,b), EP-MOF (c), and EP-EnMOF (d) coatings.

Adding UiO-66-NH2 and encapsulating UiO-66-NH2 eliminated the micrometric defects in the epoxy coating (Figure 8c,d, respectively). The observed enhancement is most likely attributed to the chemical bonds formed between the amine groups in the MOFs and the epoxide groups of the coating matrix. This chemical interaction enhances the structural resistance of the coating against defect formation by increasing its cross-linking density. The FTIR analysis was used to investigate this issue. Since signal intensity in the FTIR method depends on concentration, the MOF concentration in the samples used for FTIR analysis was 10 times higher than that of the EP-MOF coating (5 wt %). As shown in the FTIR spectrum (Figure 8), the absorption band corresponding to the epoxy ring in the epoxy resin at around 915 cm^–1^ ^33^ disappeared after the addition of UiO-66-NH2 and is no longer visible in the FTIR spectrum of the epoxy composite. Furthermore, the IR band corresponding to the primary amine at around 1650 cm^–1^ ^34^ in the FTIR spectrum of UiO-66-NH2 also disappeared due to its interaction with the epoxide groups, and it is absent in the FTIR spectrum of the epoxy composite. These results confirm the expected chemical interaction between the terminal amine groups in UiO-66-NH2 and the epoxide groups in the epoxy resin.

**Figure 8:**
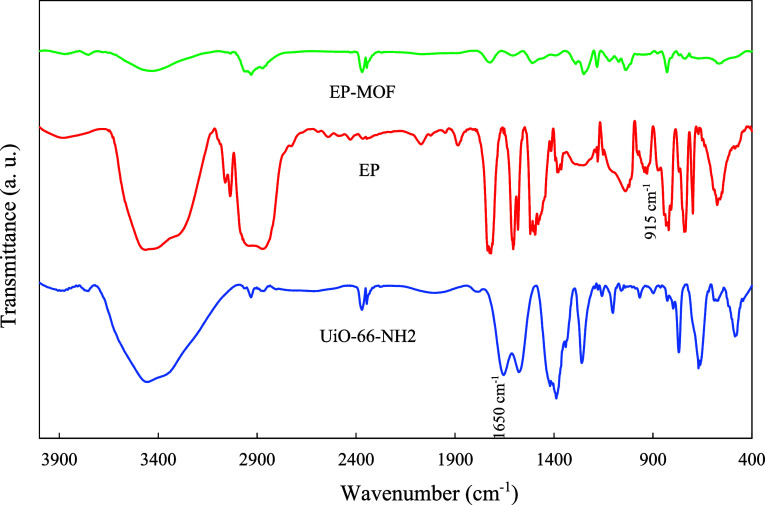
FTIR spectra of UiO-66-NH2, EP, and EP-MOF coatings.

Homogeneous dispersion of nanoparticles within anticorrosion coatings is essential. If the added particles aggregate, they can weaken the coating’s cohesion at those sites, creating potential pathways for corrosive electrolytes to penetrate. In such cases, rather than improving corrosion resistance, the nanoparticles may have detrimental effects. To examine the distribution of MOFs within the epoxy matrix, thin films extracted from the coatings were subjected to TEM analysis. Figure 9 demonstrates the uniform dispersion of the UiO-66-NH2 particles within the epoxy coating. In the low-magnification image (Figure 9a), clusters of added particles are visible throughout the matrix. At higher magnification (Figure 9b), these micrometer clusters are seen to consist of numerous nanometer MOF particles, loosely bound together. The particles exhibit a greater affinity for interaction with the epoxy coating matrix than with one another. A similar dispersion pattern was observed for the encapsulated particles, as shown in Figure 9c,d.

**Figure 9:**
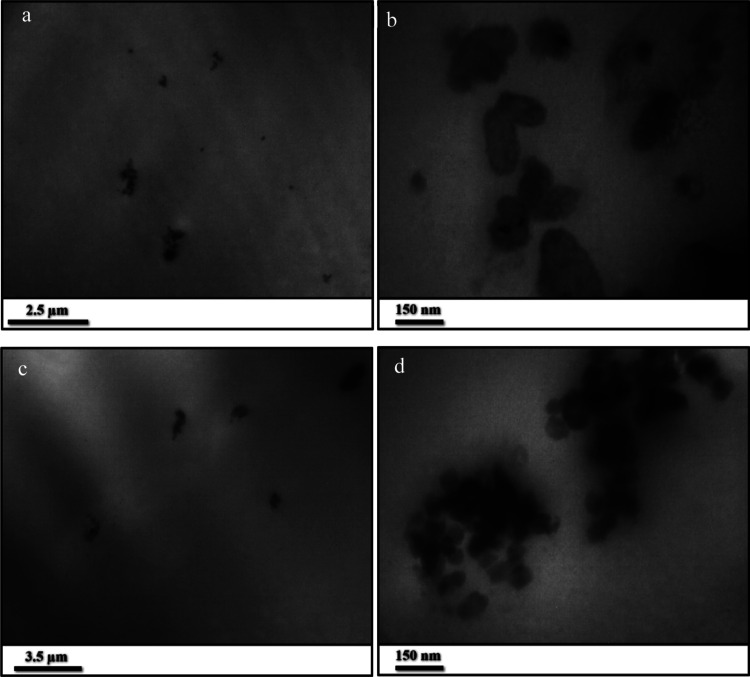
TEM images of thin films of EP-MOF (a,b) and EP-EnMOF (c,d) epoxy coatings, prepared using electromicrotomy.

The 3D AFM images of the EP, EP-MOF, and EP-EnMOF coatings are shown in Figure 10. The EP coating exhibits noticeable pores and irregularities (Figure 10a), which are substantially reduced in the composite coatings (Figure 10b,c), resulting in decreased surface roughness (*S*_a_) from 3.909 ± 0.606 to 3.260 ± 0.585 and further to 1.851 ± 0.289 μm after incorporating UiO-66-NH2 and its encapsulated form, respectively. This improvement in the EP-MOF coating is attributed to the chemical bonding of the terminal amines of MOFs and the epoxy matrix, which increases the cross-linking density, effectively removing the pores. Additionally, the encapsulation of UiO-66-NH2 ensured a uniform distribution of MOFs within the epoxy matrix, enhancing the overall smoothness of the EP-EnMOF coating. The sequential layering of charged species on the surface of encapsulated MOFs induces electrostatic repulsion, preventing their aggregation and ensuring a more uniform distribution.

**Figure 10:**
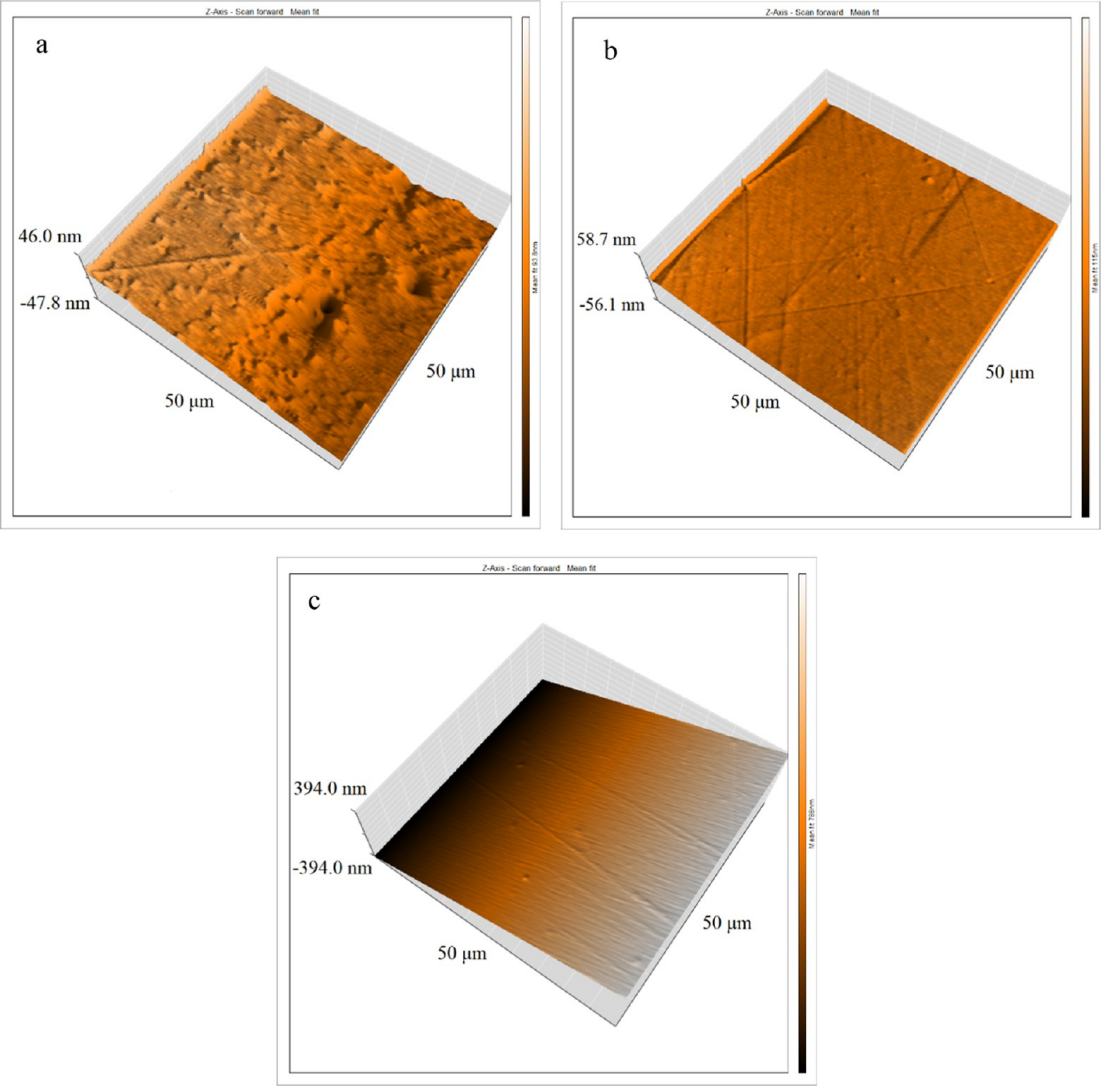
3D AFM images of the EP (a), EP-MOF (b), and EP-EnMOF (c) coatings.

The incorporation of 0.5 wt % UiO-66-NH2, which contains terminal amine groups, reduced the water contact angle of the epoxy anticorrosive coating from about 79.2 ± 2.5° to approximately 71.2 ± 1.2°. This suggests an enhancement in the coating’s hydrophilicity due to the amine groups.^35^ On the other hand, the incorporation of 0.5 wt % encapsulated UiO-66-NH2 yielded a contact angle of approximately 75.5 ± 1.3°. This intermediate contact angle suggests that encapsulation partially counteracted the MOF-induced increase in hydrophilicity, likely due to the polyelectrolyte layers, thereby balancing the surface properties (Figure 11).

**Figure 11:**
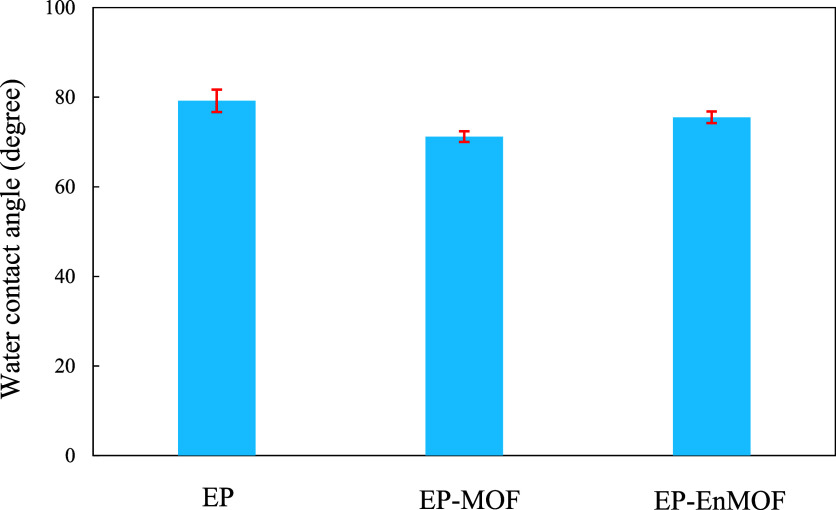
Water contact angles of the applied epoxy coatings.

### 3.3 Corrosion Studies

The impedance response of all coatings in 0.2 M HCl, as illustrated in 3D Nyquist, Bode modulus, and phase Bode plots (Figures 12, 13, 14), revealed two capacitive time constants: one at high frequency and the other at low frequency. To minimize overlap between EIS plots at different immersion times, they were presented in the 3D format. Additionally, conventional 2D Nyquist plots are provided in the Supporting Information (Figure S6). These time constants correspond to the coating itself and the charge transfer process at the metal-coating interface, respectively.^36,37^ Each capacitive time constant can be modeled by a parallel combination of a resistance and a nonideal capacitance, commonly referred to as a constant phase element (CPE). Capacitive elements in real coating systems often exhibit nonideal behavior due to surface heterogeneity, porosity, compositional heterogeneity, etc., leading to frequency-dependent capacitance. To account for this, the CPE is used in equivalent circuit models.^38,39^ The impedance of the CPE element can be expressed as follows^40^

3 $$Z_{CPE}=\frac{1}{Q{\left(j\omega \right)}^{n}}$$

Here, *j* represents an imaginary number, ω is the angular frequency, and *n* is a dimensionless parameter ranging from 0 to 1, representing the deviation from ideal capacitor behavior. When n equals 1, it signifies ideal capacitive behavior. Also, *Q* is a constant with units of *s* ^*n*^Ω^–1^ cm^–2^, representing capacitance when *n* equals 1.

**Figure 12:**
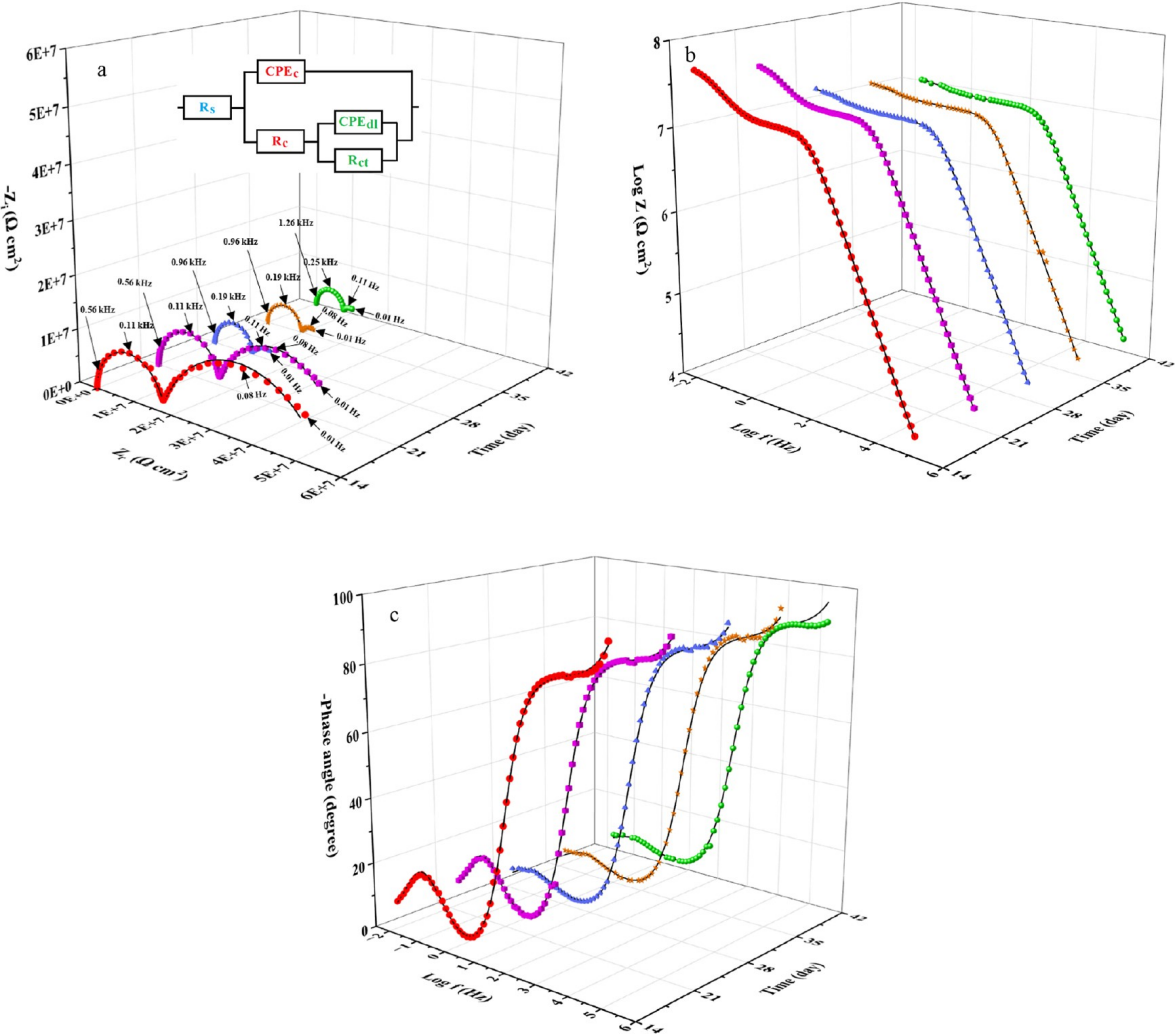
3D Nyquist (a) and Bode (b: modulus and c: phase) diagrams of the EP coating following various immersion durations in 0.2 M HCl.

**Figure 13:**
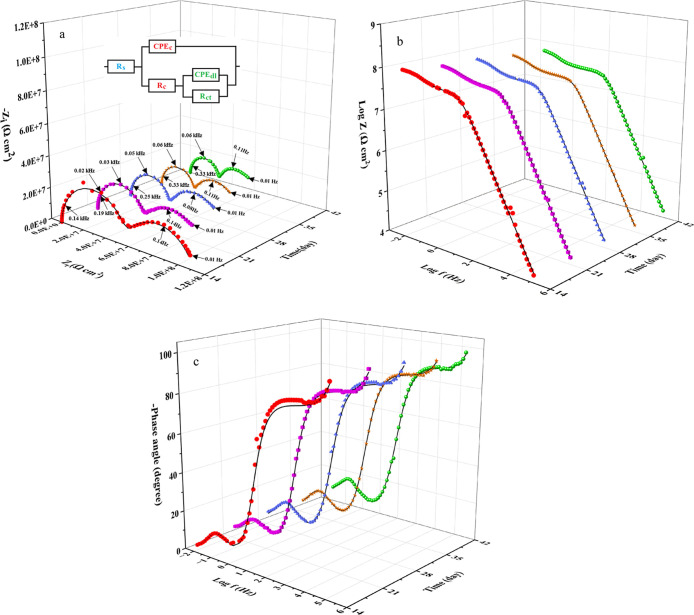
3D Nyquist (a) and Bode (b: modulus and c: phase) diagrams of the EP-MOF coating following various immersion durations in 0.2 M HCl.

**Figure 14:**
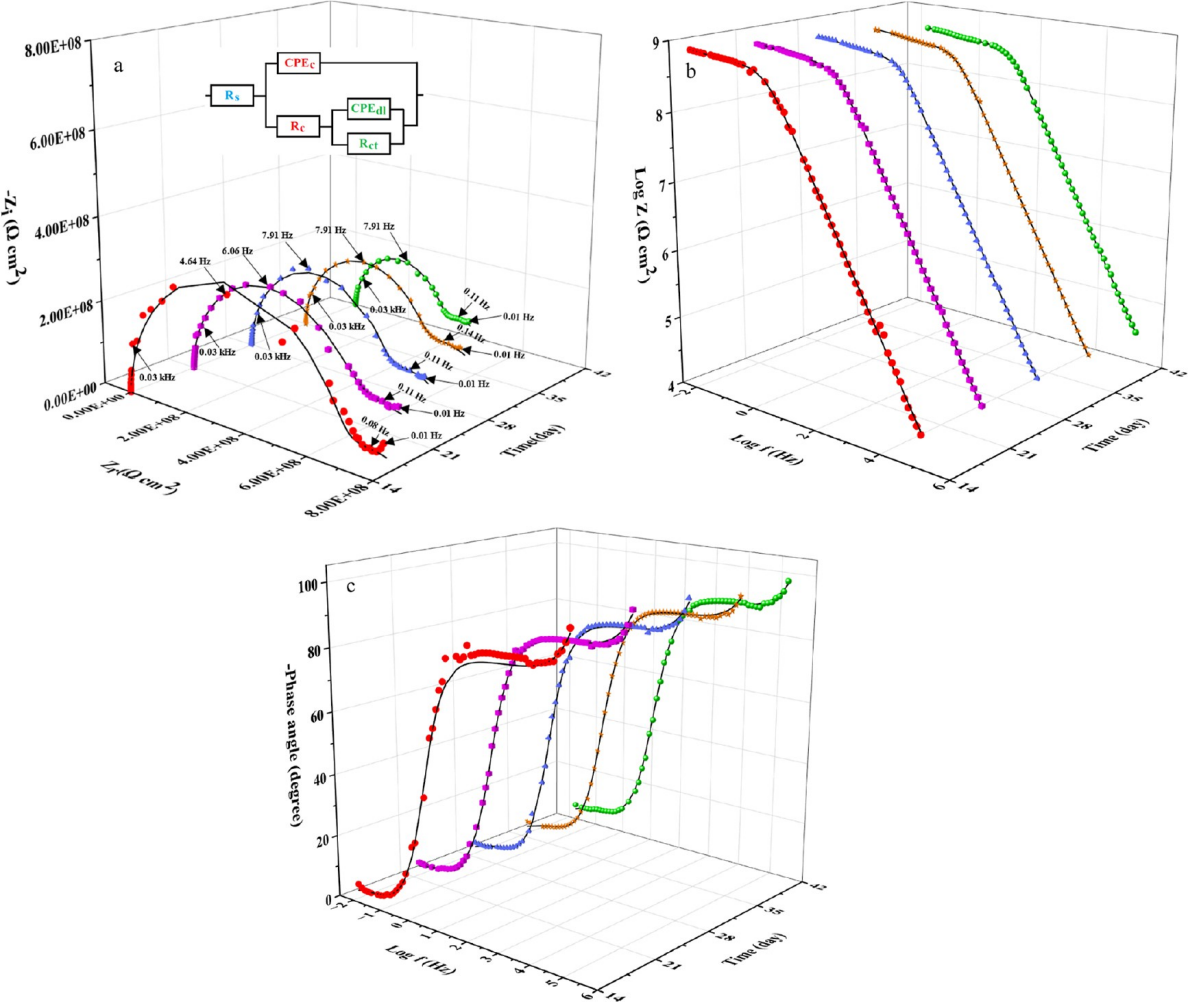
3D Nyquist (a) and Bode (b: modulus and c: phase) diagrams of the EP-EnMOF coating following various immersion durations in 0.2 M HCl.

The capacitive behavior of the coatings originates from their dielectric properties. The coating nonideal capacitance (CPE_c_) arises from the ability of the dielectric layer to store electrical charge, influenced by factors such as thickness, porosity, and microstructural features. The electrical resistance of the coating (*R*_c_) originates from its ability to impede electrical current flow and is mainly influenced by the composition, thickness, and porosity. At the metal-coating interface, an electrical double-layer capacitor develops when the charged metal interacts with the infiltrating corrosive solution. To account for the nonideal capacitance of the double layer, the CPE_dl_ is an appropriate modeling element. The deviation from ideal behavior in the electrical double layer is largely attributed to variations in the metal surface topography, which create irregularities and cause variations in the capacitance. The possible pores of the coatings also allow electrolyte penetration, leading to multiple interfacial regions with different capacitances and nonideal capacitive behavior. The charge transfer resistance (*R*_ct_), influenced by the kinetics of corrosion (oxidation–reduction) reactions at the metal-coating interface. When the corrosive solution resistance element (*R*_s_) is connected in series with the two capacitor/resistor parallel networks described, the resulting electrical equivalent circuit effectively models the recorded EIS data. The described equivalent circuit is shown in the 3D Nyquist plots.^41^ In the EIS measurements, the capacitive semicircles with smaller time constants (τ = RC) appear at higher frequencies on the left side of the Nyquist plots. So, the semicircles appearing on the left side of the recorded Nyquist diagrams are related to the epoxy coating because the epoxy coating has a much lower capacitance than the electric double layer. The experimental EIS diagrams for EP, EP-MOF, and EP-EnMOF coatings were fitted with the described equivalent circuits using Zview2 software. The impedance parameters obtained from the analysis are summarized in Table 2.

**Table 2:** EIS Parameters of the EP, EP-MOF, and EP-EnMOF Coatings after Different Immersion Times in 0.2 M HCl

sample	time (days)	*E*_OCP_ (V)	*Q*_c_ (ns^n^Ω^–1^ cm^–2^)	*n*_c_	*R*_c_ (MΩ cm^2^)	*Q*_dl_ (μs^n^Ω^–1^ cm^–2^)	*n*_dl_	*R*_ct_ (MΩ cm^2^)	*R*_p_ (MΩ cm^2^)	Chi-square
EP	14	–0.678 ± 0.005	0.126 ± 0.027	0.975 ± 0.003	14.04 ± 7.11	0.079 ± 0.039	0.608 ± 0.139	22.89 ± 9.22	36.93 ± 14.51	0.0014 ± 0.0003
EP-MOF		–0.680 ± 0.008	0.093 ± 0.015	0.950 ± 0.004	32.45 ± 17.98	0.018 ± 0.002	0.857 ± 0.072	40.32 ± 12.80	72.77 ± 29.47	0.0590 ± 0.0138
EP-EnMOF		–0.653 ± 0.018	0.063 ± 0.006	0.960 ± 0.003	527.03 ± 130.62	0.015 ± 0.009	0.631 ± 0.125	238.07 ± 46.78	765.10 ± 119.30	0.0608 ± 0.0091
EP	21	–0.678 ± 0.005	0.129 ± 0.028	0.974 ± 0.005	13.02 ± 6.30	0.138 ± 0.006	0.636 ± 0.115	15.65 ± 7.40	28.67 ± 12.00	0.0022 ± 0.0012
EP-MOF		–0.667 ± 0.013	0.088 ± 0.011	0.966 ± 0.005	21.84 ± 14.81	0.048 ± 0.019	0.789 ± 0.068	28.65 ± 14.32	50.48 ± 29.12	0.0021 ± 0.0013
EP-EnMOF		–0.664 ± 0.016	0.059 ± 0.007	0.975 ± 0.001	341.50 ± 92.00	0.011 ± 0.008	0.576 ± 0.159	302.27 ± 65.96	643.77 ± 157.82	0.0057 ± 0.0035
EP	28	–0.690 ± 0.001	0.147 ± 0.034	0.966 ± 0.010	7.33 ± 3.14	0.388 ± 0.134	0.517 ± 0.144	7.54 ± 4.72	14.87 ± 7.27	0.0034 ± 0.0036
EP-MOF		–0.666 ± 0.012	0.092 ± 0.012	0.959 ± 0.005	18.90 ± 12.64	0.074 ± 0.033	0.801 ± 0.033	25.34 ± 12.91	44.24 ± 25.54	0.0059 ± 0.0058
EP-EnMOF		–0.672 ± 0.014	0.060 ± 0.008	0.973 ± 0.002	311.30 ± 112.48	0.018 ± 0.014	0.672 ± 0.030	172.86 ± 58.50	484.16 ± 138.96	0.0042 ± 0.0018
EP	35	–0.703 ± 0.003	0.155 ± 0.035	0.960 ± 0.006	7.13 ± 5.13	0.426 ± 0.149	0.584 ± 0.116	4.77 ± 0.87	11.90 ± 5.90	0.0120 ± 0.0066
EP-MOF		–0.664 ± 0.011	0.093 ± 0.009	0.962 ± 0.008	15.90 ± 10.68	0.098 ± 0.050	0.798 ± 0.038	20.02 ± 11.77	35.92 ± 22.45	0.0015 ± 0.0008
EP-EnMOF		–0.671 ± 0.016	0.061 ± 0.010	0.974 ± 0.005	250.53 ± 117.94	0.024 ± 0.023	0.656 ± 0.055	155.83 ± 61.12	406.36 ± 163.57	0.0030 ± 0.0022
EP	42	–0.666 ± 0.022	0.157 ± 0.035	0.968 ± 0.006	4.98 ± 3.27	0.722 ± 0.222	0.529 ± 0.151	2.97 ± 0.48	7.95 ± 3.73	0.0019 ± 0.0015
EP-MOF		–0.673 ± 0.014	0.101 ± 0.008	0.953 ± 0.009	14.78 ± 9.16	0.132 ± 0.065	0.816 ± 0.033	17.80 ± 9.39	32.58 ± 18.56	0.0065 ± 0.0036
EP-EnMOF		–0.661 ± 0.024	0.067 ± 0.012	0.965 ± 0.006	206.83 ± 82.09	0.026 ± 0.023	0.659 ± 0.055	120.50 ± 58.40	327.33 ± 117.40	0.0048 ± 0.0020

When epoxy coatings are immersed in a neutral corrosive solution such as 3.5 wt % NaCl, three distinct phases in polarization resistance (*R*_p_ = *R*_c_ + *R*_ct_) changes occur over time. Initially, a decrease occurs in the first few days as the corrosive solution begins to penetrate toward the substrate. This is typically followed by a transient increase during the intermediate phase, which is attributed to the accumulation of corrosion products within the pores. Finally, during prolonged immersion, further decline sets in due to the dissolution of these corrosion products, which previously filled the pores, allowing greater quantities of the corrosive solution to enter.^42,43^ For the epoxy coatings applied in this study, where corrosion tests were conducted in 0.2 M HCl, a different trend in the *R*_p_ variation was observed over time. As immersion progressed, the polarization resistance declined progressively due to the corrosive solution penetrating all three applied coatings. The lack of a transient *R*_p_ rise is likely due to the acidity of the test solution, which hinders the formation of solid corrosion products inside the pores. The progressive *R*_p_ decrease was accompanied by a steady rise in the capacitance of both the epoxy matrix and the electrical double layer (Table 2). This phenomenon verifies the progressive infiltration of the aqueous corrosive solution into the coating, eventually reaching the metal-coating interface. Water, with its high dielectric constant of approximately 78.3 at 25 °C, enhances capacitance values as it infiltrates the coating and accumulates at the interface zone.^44,45^

While the polarization resistance of all three coatings progressively declined during extended immersion in the corrosive solution, distinct differences remain in the parameters influencing their corrosion resistance. For instance, the coating resistance and charge transfer resistance of the EP coating showed significant increases throughout all immersion durations following the addition of UiO-66-NH2. By the second week of immersion, the *R*_c_ and *R*_ct_ of the EP coating measured 14.04 and 22.89 MΩ cm^2^, respectively. After UiO-66-NH2, the values increased, reaching 32.45 and 40.32 MΩ cm^2^, respectively. The difference in *R*_c_ and *R*_ct_ between the coatings persisted even after 6 weeks of immersion in the corrosive medium. The *R*_c_ and *R*_ct_ of the EP coating increased from 4.98 and 2.97 to 14.78 and 17.80 MΩ cm^2^, respectively. Simultaneously, a significant reduction in *Q*_c_ and *Q*_dl_ of the EP coating was observed over the entire immersion period following the addition of UiO-66-NH2, suggesting a decreased coating permeability to the corrosive solution (Table 1). The observed changes suggest enhanced corrosion resistance of the epoxy coating after the incorporation of UiO-66-NH2, due to several contributing factors. One possible reason is the filling of the coating’s pores with MOFs, which creates winding pathways for the corrosive electrolyte. This reduces the amount of infiltrated electrolyte and slows its penetration toward the steel surface. Additionally, the porous structure of the MOFs may absorb some of the infiltrated electrolyte and hinder its access to the alloy surface. Another crucial factor is the potential chemical interaction between the terminal amine groups of UiO-66-NH2 and the functional epoxide groups in the coating. This interaction enhances the structural strength of the coating in areas with low cross-linking density, prevents defect formation, and improves its impermeability.^46^ A schematic of the interaction between the aforementioned functional groups is shown below (Scheme 3).

**Scheme 3:**
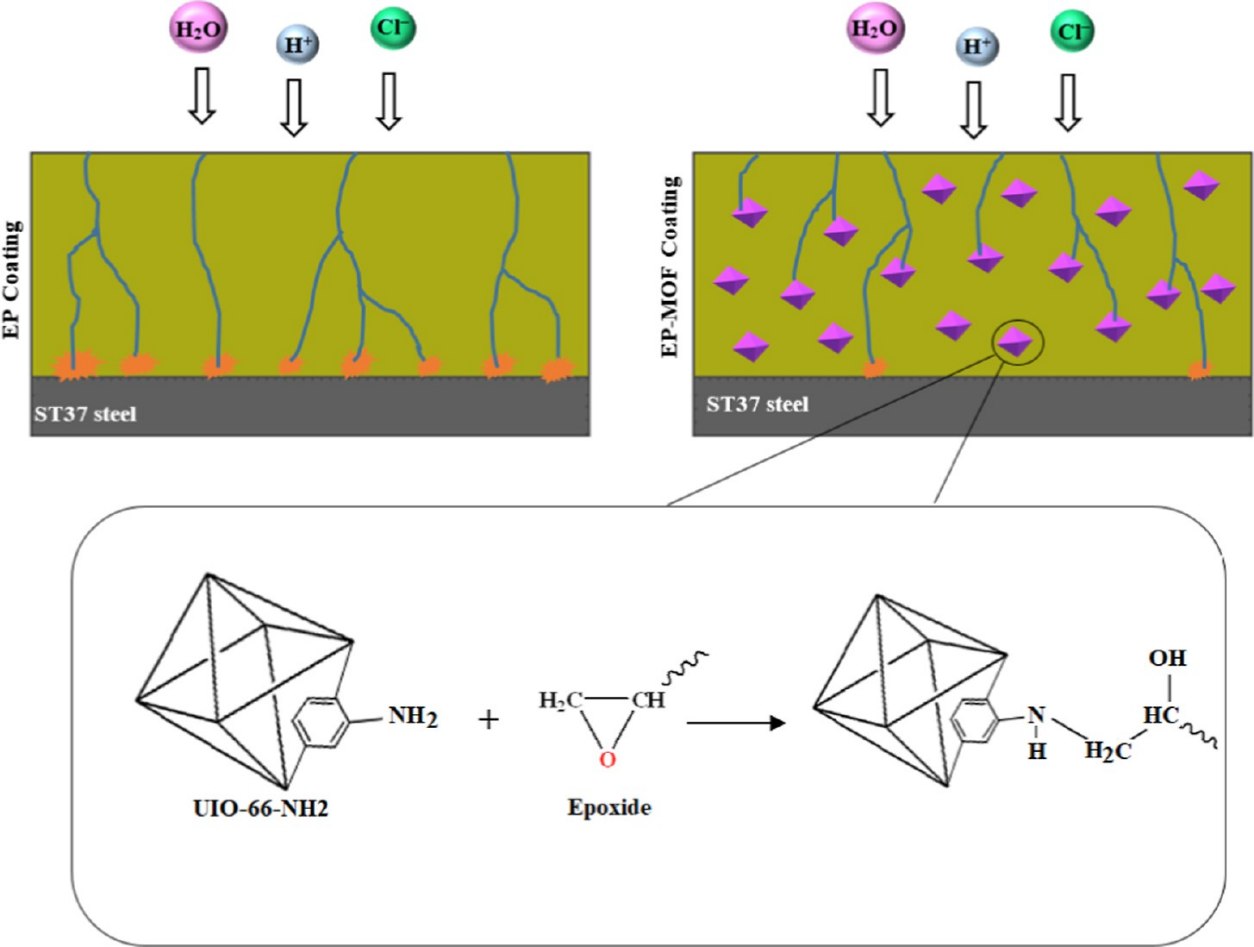
Interaction between Amine and Epoxide Groups within the EP-MOF Coating Matrix

Moreover, the incorporation of 0.5 wt % encapsulated UiO-66-NH2 led to significant changes in the impedance characteristics of the EP coating. Notably, substantial increases in *R*_c_, *R*_ct_, and *R*_p_ were observed compared to both EP and EP-MOF coatings, confirming a substantial improvement in anticorrosion performance. A consistent enhancement in anticorrosion performance was maintained throughout the immersion period. For example, after 6 weeks, the *R*_c_, *R*_ct_, and *R*_p_ of the EP-EnMOF coating reached approximately 206.83, 120.50, and 327.33 MΩ cm^2^, respectively. The values were approximately 41, 40, and 41 times higher than those of the EP coating and roughly 14, 7, and 10 times greater than those of the EP-MOF coating, respectively. Simultaneously, a substantial reduction in *Q*_c_ and *Q*_dl_ was observed in the epoxy coating following the addition of encapsulated UiO-66-NH2 (Table 2). These changes further indicate a substantial advancement in corrosion protection. The improved anticorrosion features of the EP coating following the addition of encapsulated UiO-66-NH2 are not solely due to the chemical interaction between MOFs and the epoxy matrix. This is because most terminal amine groups are shielded by the polyelectrolyte shell, preventing direct interaction with the epoxy matrix. Additional mechanisms contributing to corrosion protection may include the filling of potential pores and the formation of tortuous pathways for the corrosive electrolyte. However, the substantial enhancement in corrosion protection of the EP-EnMOF coating cannot be entirely explained by these mechanisms. Instead, it appears that the primary factor is the controlled release of Schiff base molecules from the pores of UiO-66-NH2, followed by their adsorption onto the metal surface. Upon penetration of the corrosive electrolyte into the coating and its contact with the metal surface, corrosion reactions (metal oxidation and water reduction) are initiated, resulting in localized acidic and alkaline pH shifts at anodic and cathodic sites, respectively. Under these conditions, the polyelectrolyte shell opens, allowing the gradual release of Schiff base molecules from the MOF pores, which then adsorb onto the metal surface. As confirmed by UV–visible studies, the most significant release occurs under alkaline conditions. Therefore, it can be expected that the Schiff base molecules are predominantly released near the local cathodic sites. Upon release, the Schiff base molecules are expected to adsorb onto the active corrosion sites, thereby inhibiting further corrosion. The smart release and adsorption process of the Schiff base molecules is schematically illustrated in Scheme 4.

**Scheme 4:**
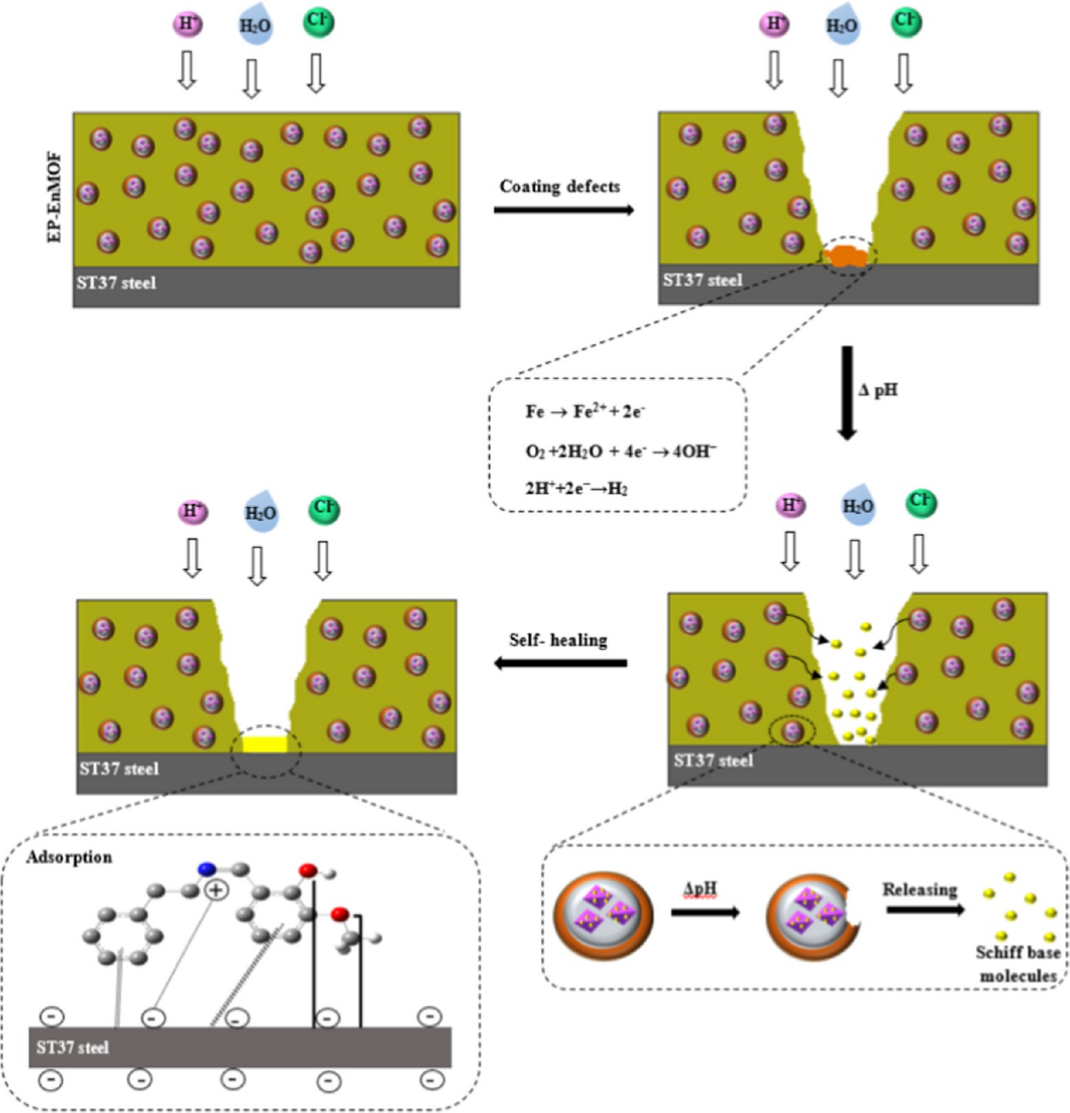
Intelligent Release of Schiff Base Molecules at Corrosion-Damaged Sites and Their Subsequent Adsorption

The observed capacitance variations validate the adsorption of the Schiff base inhibitor onto the steel. As noted, the *Q*_c_ and *Q*_dl_ values of the EP-EnMOF coating remain lower than those of the EP and EP-MOF coatings throughout the immersion period. Notably, capacitance reduction is more pronounced in the electrical double layer (*Q*_dl_) than in the epoxy layer (*Q*_c_). For instance, the *Q*_dl_ value of the EP-EnMOF coating decreases by over 27 times compared to the EP coating after 6 weeks of immersion, while the *Q*_c_ parameter declines just over two times. This sharp decline in *Q*_dl_ stems from the release and adsorption of the Schiff base molecules onto the metal surface, a replacement process where inhibitor molecules substitute preadsorbed water molecules. Replacing adsorbed water molecules, which have a high dielectric constant, with organic Schiff base molecules leads to a reduction in the *Q*_dl_. The adsorption of larger Schiff base molecules contributes to the thickening of the electrical double layer, thereby reducing capacitance.^47,48^ After 2 weeks of immersion, the *Q*_dl_ value for EP-EnMOF is reduced by 5.2 times compared to the EP coating, progressively increasing to over 27 times by week six, highlighting the gradual inhibitor release and adsorption at anodic and cathodic active sites.

Given the innovative nature of the synthesized Schiff base and the lack of prior studies on its effectiveness as a corrosion inhibitor, its adsorption mechanism on steel will be investigated using DFT in a separate section.

Since the PDP method is inherently destructive, conducting it at different immersion times could alter the properties of the applied coating. Therefore, these analyses were performed exclusively after the 6 week immersion period, with the corresponding graphs presented in Figure 15. The recorded curves were analyzed using the Tafel extrapolation method. The results, including *E*_corr_, *R*_p_, anodic and cathodic Tafel slopes (*b*_a_ and b_c_), and corrosion current density (*J*_corr_), are presented in Table 3.

**Figure 15:**
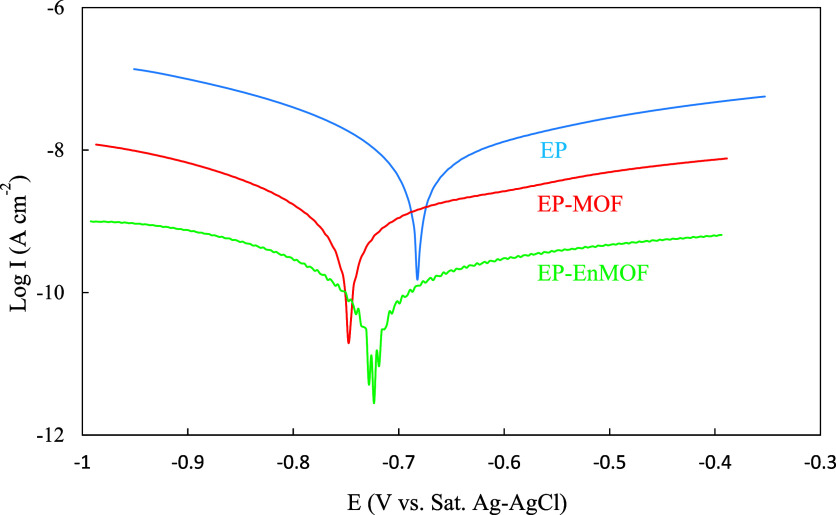
Potentiodynamic polarization curves of the EP, EP-MOF, and EP-EnMOF coatings after 6 weeks of immersion in 0.2 M HCl.

**Table 3:** Polarization Parameters of the EP, EP-MOF, and EP-EnMOF Coatings after 6 weeks of Immersion in 0.2 M HCl

coating	*E*_corr_ (V vs Sat. Ag–AgCl)	*R*_p_ (MΩ cm^2^)	*b*_a_ (mV dec^–1^)	*b*_c_ (mV dec^–1^)	*J*_corr_ (nA cm^–2^)
Ep	–0.681 ± 0.008	9.58 ± 4.28	128.50 ± 5.42	196.66 ± 2.65	4.32 ± 1.83
Ep-MOF	–0.749 ± 0.003	45.33 ± 6.94	105.78 ± 11.10	169.85 ± 8.41	0.64 ± 0.12
Ep-EnMOF	–0.727 ± 0.002	533.22 ± 164.66	143.57 ± 17.35	176.43 ± 8.41	0.06 ± 0.01

A comparison of the polarization resistance values calculated using the polarization method after 6 weeks of immersion with those obtained via the impedance method at the same immersion time (Tables 2 and 3) revealed some numerical discrepancies, which are expected given the inherent differences between the two techniques. However, the numerical variation is relatively minor, and importantly, there is strong agreement in the overall trend between the results of both methods. For instance, after 6 weeks of immersion, the polarization resistance of the EP-MOF coating measured by EIS is approximately 41 times higher than that of the EP coating, whereas the increase measured by polarization is approximately 55 times. Thus, the polarization measurement results validated the findings obtained through the EIS analysis. Furthermore, the corrosion current density values showed a significant decrease after MOF incorporation, particularly encapsulated MOFs, highlighting an enhancement in corrosion resistance. Typically, a decrease in the corrosion current of coated metal samples corresponds to a shift in the corrosion potential toward more positive values. However, it is important to note that corrosion potential is a thermodynamic parameter, and its variations do not always directly reflect changes in the coating’s corrosion behavior. As shown in Table 3, despite the significant reduction in the corrosion current density of the epoxy coating after MOF incorporation, the nanocomposite coatings exhibited a more negative corrosion potential. These shifts may result from interactions at the coating-metal interface, including the accumulation of corrosion products, adsorption of corrosion inhibitors, local pH changes, and other related factors.

Direct numerical comparison of corrosion-related electrochemical test results with published studies remains inherently challenging, given the sensitivity of corrosion parameters to several factors such as the metallic substrate type, properties of the epoxy resin and curing agent, coating thickness, test environment, immersion duration, etc. Despite these complexities, Table 4 presents a comparative assessment of the degree of corrosion resistance improvement achieved in the current study. These results are compared with findings from several analogous studies cited in the literature.^8,49–51^ Notably, the present findings demonstrate remarkable corrosion protection performance relative to that of the referenced investigations.

**Table 4:** Comparison of Corrosion Resistance Enhancement in the Current Study versus Similar Published Research

refs	coating substrate	coating thickness (μm)	nanocarrier	inhibitor	concentration of inhibitor-loaded nanocarriers	parameter indicating corrosion resistance	test environment	immersio*n* time	results
^8^	epoxy steel	50 ± 2	reduced graphene oxide@Silica nanoparticle	benzotriazole	0.25 wt.% relative toresin	|Z|_*f*=0.01 Hz_		90 days	≈7.7 times improvement (from 70.123 to 538.12 MΩ cm^2^
 ^49^	epoxy- P110 steel	60 ± 10	polydopamine-functionalized Ti_3_C_2_ MXene-doped ZnAl LDH	molybdate	1 wt.% relative to the resin + hardener mixture	*R*_c_ + *R*_ct_	3.5 wt % NaCl	10 days	≈21.5 times improvement (from 7.92 to 170 MΩ cm^2^)
^50^	epoxy-ST37	45 ± 4	cellulose nanocrystals	losartan potassium	1 wt.% relative to the resin + hardener mixture	*R*_f_ + *R*_ct_	0.1 HCl	7 days	≈5 times improvement (from 0.658 to 3.272 MΩ cm^2^)
^51^	epoxy- Q235 carbon	30 ± 3	ZnAl LDH@ZIF-8	1H-benzotriazole and molybdate	0.5 wt % relative to the resin + hardener mixture	|Z|_*f*=0.01 Hz_	3.5 wt % NaCl	42 days	≈27 times improvement (from 158 to 4240 MΩ cm^2^)
^52^	epoxy carbon steel	30 ± 1	alkyl quaternary ammonium salt-modified MMT (AA-MMT)	8-hydroxy quinoline	3 wt % relative to the resin + hardener mixture	|Z|_*f*=0.01 Hz_	0.5 M NaCl	21 days	AA-MMT + Epoxy ≈1 × 10^7^ Ω cm^2^ and 8HQ@MMT + Epoxy ≈1–2 × 10^8^ Ω cm^2^ pure epoxy/Not reported
recent work	epoxy-ST37 steel	70 ± 1	UiO-66-NH2	2-methoxy-6-((phenethylimino)methyl)phenol	0.5 wt % relative to the resin + hardener mixture	*R*_c_ + *R*_ct_	0.2 M HCl	42 days	≈41 times improvement (from 7.95 to 327.33 MΩ cm^2^)

### 3.4 Morphological Studies after the Corrosion Tests

Figure 16 presents digital photographs of the coated samples taken after immersion in the corrosive solution. The EP coating exhibits severe corrosion effects. Red iron rust is visible almost everywhere in the sample. Due to the infiltration of the corrosive acid solution, severe corrosion has occurred beneath the coating, with corrosion products accumulating through its pores. In the case of the sample containing UiO-66-NH2, the corrosion intensity is much lower. Also, apart from a red stain, there are almost no visible corrosion traces in the EP-EnMOF sample, indicating its excellent corrosion resistance in a highly corrosive acidic solution after a long immersion time.

**Figure 16:**
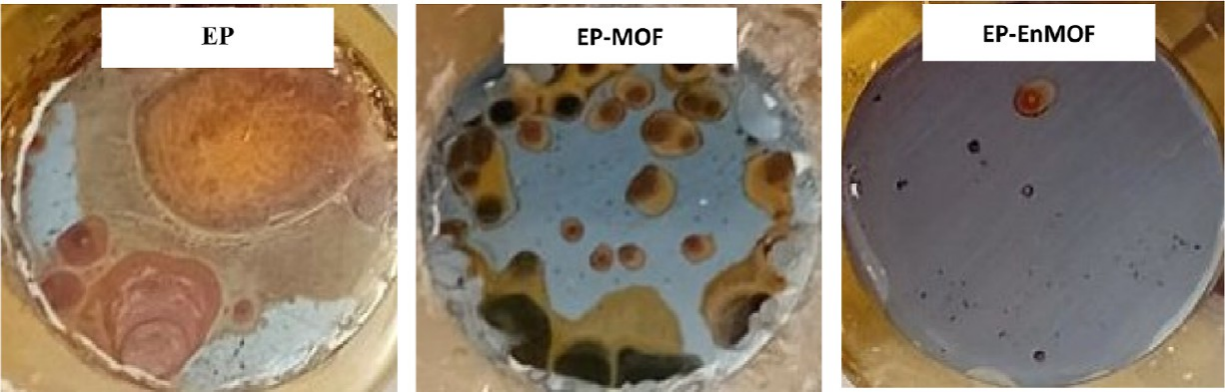
Visual images of the EP, EP-MOF, and EP-EnMOF coatings upon completion of the corrosion tests.

To better analyze the surface morphology after immersion in the corrosive medium, we captured microscopic images. The EP sample exhibited the formation of microscopic holes, signifying severe corrosion across the surface (Figure 17a). In contrast, the EP-MOF sample displayed a notable reduction in severe corrosion effects compared to that of the EP sample. However, localized areas showed delamination of the coating and the presence of cracks. These cracks appeared shallow, not penetrating deep into the coating, and are more indicative of scaling than structural damage (Figure 17b). The EP-EnMOF coating exhibits no signs of corrosion, including pitting, accumulation of corrosion products, or delamination (Figure 17c). The obtained results are consistent with the corrosion test data.

**Figure 17:**
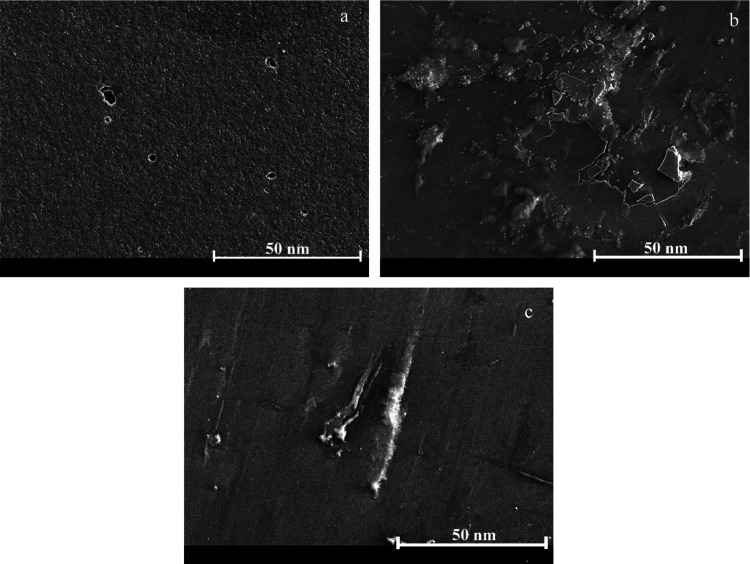
FESEM images of the EP (a), EP-MOF (b), and EP-EnMOF (c) coatings upon completion of the corrosion tests.

### 3.5 Mechanism of Inhibitor Adsorption

Quantum chemical simulations were conducted using DFT to investigate the adsorption mechanism of the delivered Schiff base molecules on the steel surface.^53–55^ The quantum chemical parameters calculated included the total energy, *E*_LUMO_ (energy of the lowest unoccupied molecular orbital), *E*_HOMO_ (energy of the highest occupied molecular orbital), band gap energy (Eg, determined as *E*_HOMO_–*E*_LUMO_), number of electrons transferred (Δ*N*), and dipole moment (μ). The energy levels of LUMO and HOMO correspond to the electron affinity (*A* = −*E*_LUMO_) and the ionization potential (*I* = −*E*_HOMO_), respectively. The absolute electronegativity (χ) and hardness (η) values for an inhibitor are related to A, I, and Δ*N* as specified by the following equations^56,57^

4 $$\chi =\frac{I+A}{2}$$

5 $$\eta =\frac{I-A}{2}$$

6 $$\Delta N=\frac{\chi_{Fe}-\chi_{inh}}{2\left(\eta_{Fe}+\eta_{inh}\right)}$$

where χ_inh_ and χ_Fe_ show the absolute electronegativity of the inhibitor and the Fe atom, respectively, also η_inh_ and η_Fe_ illustrate the absolute hardness of the Schiff base and Fe, respectively. The theoretical values of 0 for η_Fe_ and 7 eV for χ_Fe_ were utilized according to a prior study by Ebenso and co-workers.^58^

It is important to note that the ionization energy is a key factor in understanding chemical reactivity. When ionization energy is high, substances exhibit remarkable stability and minimal reactivity, whereas lower ionization energy is associated with pronounced atomic and molecular activity.^59^ Also, the molecular stability and reactivity can be effectively evaluated by using absolute hardness as a key parameter. To determine the adsorption energy (*E*_ads_) of the studied system, the following mathematical expressions were employed

7 $$E_{ads}=E_{Schiffbase-Fe}-\left(E_{Schiffbase}+E_{Fe}\right)$$

where *E*_Schiffbase‑Fe_ represents the total energy of the Schiff base-Fe system, *E*_Schiffbase_ is the energy of the Schiff base molecule, and *E*_Fe_ is the energy of the Fe atom in the liquid phase (Table 4). A negative adsorption energy signifies a thermodynamically favorable adsorption process, while a positive value implies system instability. The computed *E*_ads_ confirms the Schiff base molecule’s adsorption onto the steel surface, which occurs through both physisorption and chemisorption mechanisms.^60,61^ Furthermore, the charge analysis results showed that electrons were transferred from the d-orbitals of Fe atoms to the unoccupied antibonding orbitals of Schiff base molecules.^62–64^ The inhibitor’s ability to adsorb onto the metal surface or donate electrons is governed by the electron transfer fraction (Δ*N*). Based on the findings of Lukovits and co-workers,^65^ when Δ*N* < 3.6, the inhibition efficiency improves as the electron-donating capacity of the metal surface increases. The calculated value of this parameter was 2.305, indicating a strong adsorption capability of the Schiff base on the metal surface (Table 5).

**Table 5:** Calculated Values of Some Reactivity Indices of Pure Schiff Base and Schiff Base Fe Molecules in the Liquid Phase

system	total energy (a.u)	*E*_HOMO_ (a.u)	*E*_LUMO_ (a.u)	*E*_ads_ (eV)	*E*_g_ (a.u)	*I* (a.u)	*A* (a.u)	χ (a.u)	η (a.u)	μ (Debye)	Δ*N*
Schiff base	–825.09050	–0.12498	–0.05170		0.0732	0.12498	0.05170	0.08834	0.03664	7.4858	
Schiff base-Fe	–2088.64709	–0.12698	–0.05191	–1.679	0.0750	0.12698	0.05191	0.08944	0.03753	4.0373	2.305

The optimized structures HOMO and LUMO for the Schiff base molecule before and after interaction with the Fe atom are illustrated in Figure S10. To elucidate the mechanism behind steel corrosion inhibition in HCl solution, an adsorption and protection model was introduced, as depicted in Figure 18. The adsorption of Schiff base molecules onto the steel surface occurs through various mechanisms: (i) physisorption via electrostatic attraction between protonated inhibitor molecules and preadsorbed Cl^–^ ions, (ii) chemisorption involving interactions between lone electron pairs of heteroatoms (O, N) in the Schiff base and vacant Fe d-orbitals, and (iii) retro-donation, where π-electrons of the aromatic ring in the Schiff base engage with the empty d-orbitals of Fe atoms.

**Figure 18:**
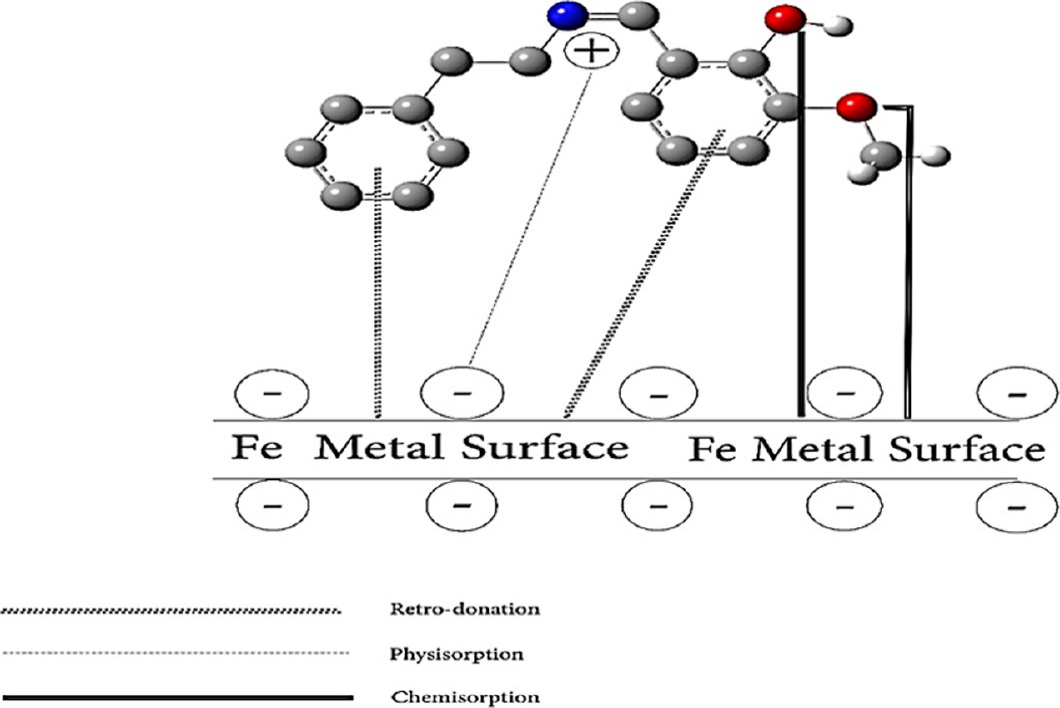
Schematic overview illustrating the adsorption interactions between Schiff base molecules and steel surfaces in an HCl environment.

## 4 Conclusion

A smart epoxy coating incorporating UiO-66-NH2 nanocarriers loaded with a newly synthesized Schiff base was successfully developed for corrosion protection of ST37 steel. Structural and morphological analyses confirmed effective Schiff base encapsulation without compromising the MOF crystallinity, with significant reductions in surface area and pore volume, validating inhibitor loading. The pH-responsive release of the Schiff base inhibitor was demonstrated via UV–vis spectroscopy with maximal release at pH 12. FTIR confirmed chemical bonding between the MOF amino groups and epoxy rings, leading to uniform MOF dispersion in the matrix. AFM analysis showed a substantial roughness reduction after incorporating UiO-66-NH2 and its encapsulation. Electrochemical studies revealed a marked improvement in corrosion resistance, with encapsulated UiO-66-NH2 yielding an *R*_p_ value of 327.33 MΩ cm^2^ (∼41-fold and ∼10-fold higher than the neat and UiO-66-NH2-filled coatings, respectively). These results were also confirmed with the PDP technique. DFT calculations corroborated Schiff base adsorption onto the steel through chemical and physical interactions. This environmentally responsive system holds promise for scalable industrial applications, such as chemical processing and water treatment infrastructure, and invites future exploration into sustainable, smart-release technologies with long-term field performance.

## Supplementary Material



## Notes

The ^1^H NMR, ^13^C NMR, FTIR, and HRMS analyses, which confirm the successful synthesis of the Schiff base compound, amount of loaded corrosion inhibitor into the nanocarriers determined by UV–visible spectroscopy to calculate both loading capacity and encapsulation efficiency, the corresponding spectrum, 2D Nyquist plots of the EP, EP-MOF, and EP-EnMOF coatings following various immersion durations in 0.2 M HCl to improve data clarity, and the optimized structures and HOMO and LUMO orbitals for pure Schiff base and Schiff base-Fe compounds in the solvated phase (PDF)
