# Supplementary material for: pH-Responsive Epoxy Coating Incorporating a Novel Schiff Base-Loaded UiO-66-NH2 with an Encapsulated Shell for Long-Term Steel Corrosion Protection
Source: ACS Omega. 2025 Nov 26;10(48):58490–513. doi: 10.1021/acsomega.5c06277 (PMC12771042; doi:10.1021/acsomega.5c06277)
Supplement: Supplementary file 1 [file ao5c06277_si_001.pdf]

Supporting information for

pH-responsive epoxy coating incorporating a novel Schiff base–loaded UiO-66-NH2 with encapsulated shell for long-term steel corrosion protection

Tahere Miri[1], Davod Seifzadeh[1, 2]*, Yunus. Kara*[2], Burak Dikici[3], Ozlem Gundogdu[2,4], Sertan Aytaç[2,4], Hadi Basharnavaz[5]

1-Department of Chemistry, University of Mohaghegh Ardabili, Ardabil 5619911367, Iran

2-Department of Chemistry, Sciences Faculty, Atatürk University, Erzurum 25240, Turkey

3- Department of Mechanical Engineering, Ataturk University, Erzurum 25240, Turkey

4-Kırşehir Ahi Evran University, Kaman Vocational School, Department of Food Technology, Kırsehir, Turkey

5- Department of Chemistry, College of Science, Yazd University, Yazd, Iran

*Corresponding author, e-mail: yukara@atauni.edu.tr / seifzadeh@uma.ac.ir

Table of Contents

1- ^{1}H NMR spectrum of 2-methoxy-6-((phenethylimino)methyl)phenol……….................................S2

2- ^{13}C NMR spectrum of 2-methoxy-6-((phenethylimino)methyl)phenol ……..…………………........S2

3- FTIR spectra of 2-methoxy-6-((phenethylimino)methyl)phenol ……………..……………………S2

4- HRMS spectra of 2-methoxy-6-((phenethylimino)methyl)phenol ……….........................………S3

5- UV-visible spectrun to determine the amount of loaded Schiff base into the pores of the UiO-66-NH2 nanocarriers …..…………………………………………………………………………..S3

6- 2D Nyquist plots of the EP coatings following various immersion durations in 0.2 M HCl………………………………………………………………………………………..S4

7- 2D Nyquist plots of the EP-MOF coatings following various immersion durations in 0.2 M HCl………………………………………………………………………………………..S4

8- 2D Nyquist plots of the EP-EnMOF coatings following various immersion durations in 0.2 M HCl……………………………………………………………………………….………S5

9- Optimized structures for pure Schiff base and Schiff base-Fe compounds in the solvated phase……………………………………………………………………………….……..S5

10- HOMO for pure Schiff base and Schiff base-Fe compounds in the solvated phase……….………..S5

11- Optimized structures (a), HOMO (b), and LUMO (c) for pure Schiff base and Schiff base-Fe compounds in the solvated phase……………………………………………………….. S6

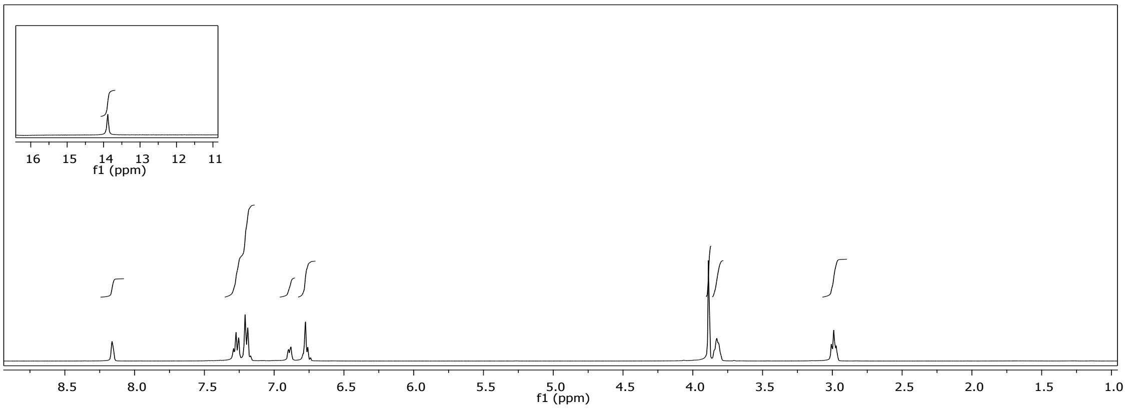

Figure S1. ^{1}H NMR spectra of the synthesized Schiff base (2-methoxy-6-((phenethylimino)methyl)phenol) compound

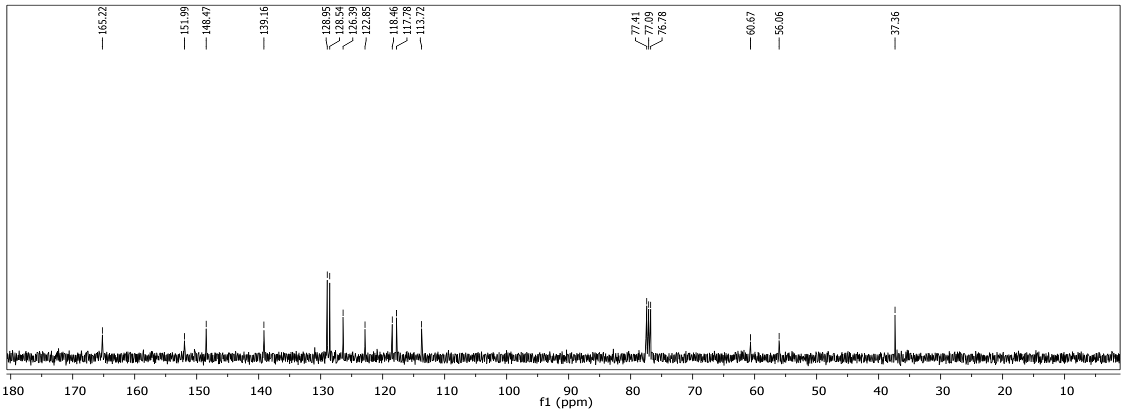

Figure S2. ^{13}C NMR spectra of the synthesized Schiff base compound

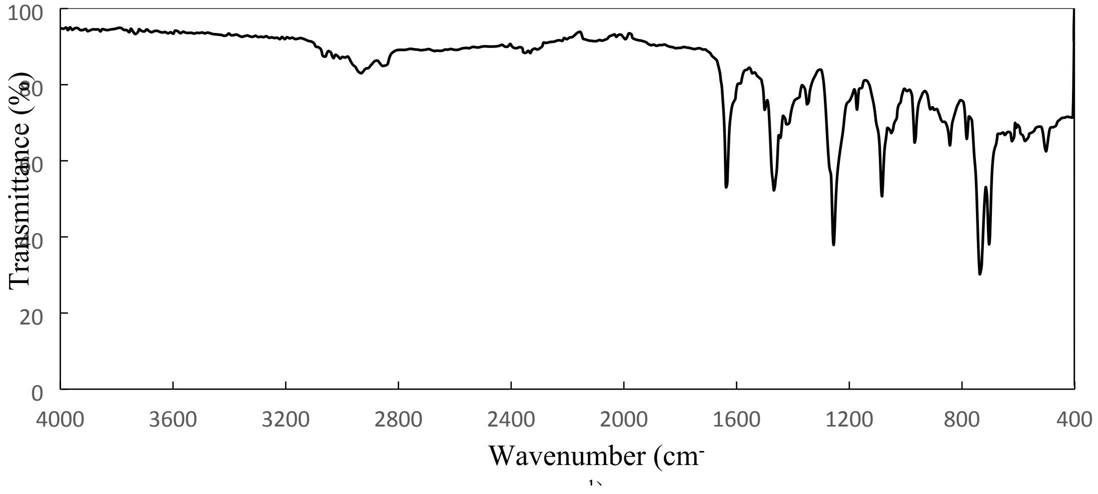

Figure S3. FTIR spectra of the synthesized Schiff base compound

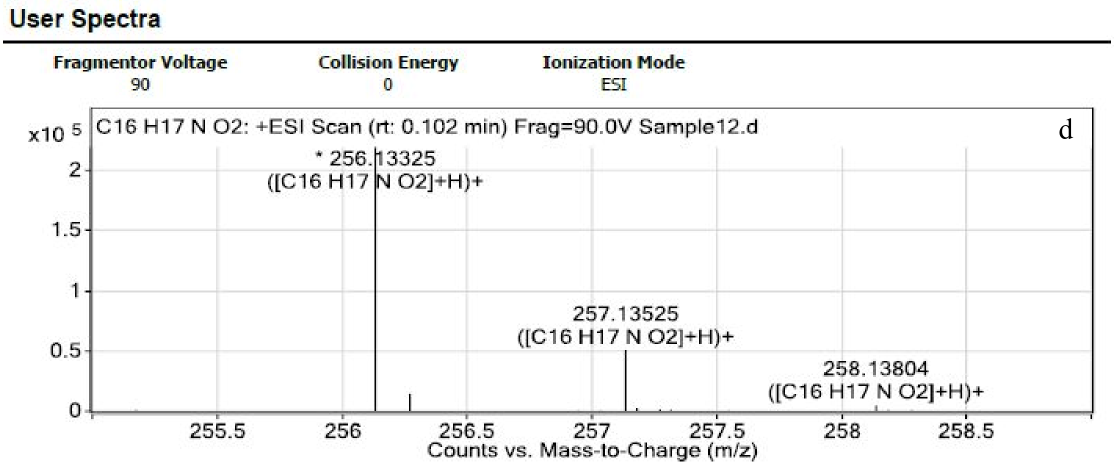

Figure S4. HRMS spectra of the synthesized Schiff base compound

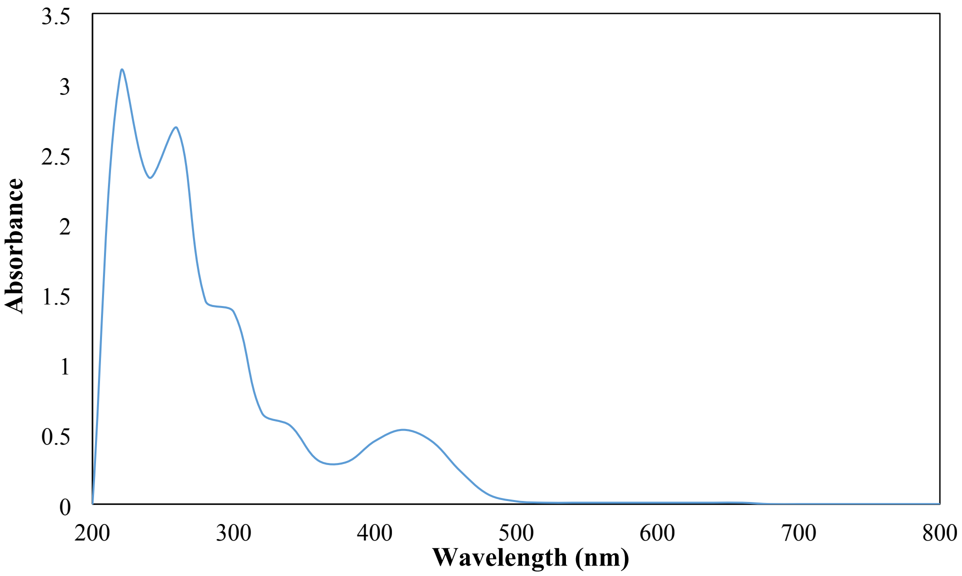

Figure S5. UV-visible spectrun to determine the amount of loaded Schiff base into the pores of the UiO-66-NH2 nanocarriers

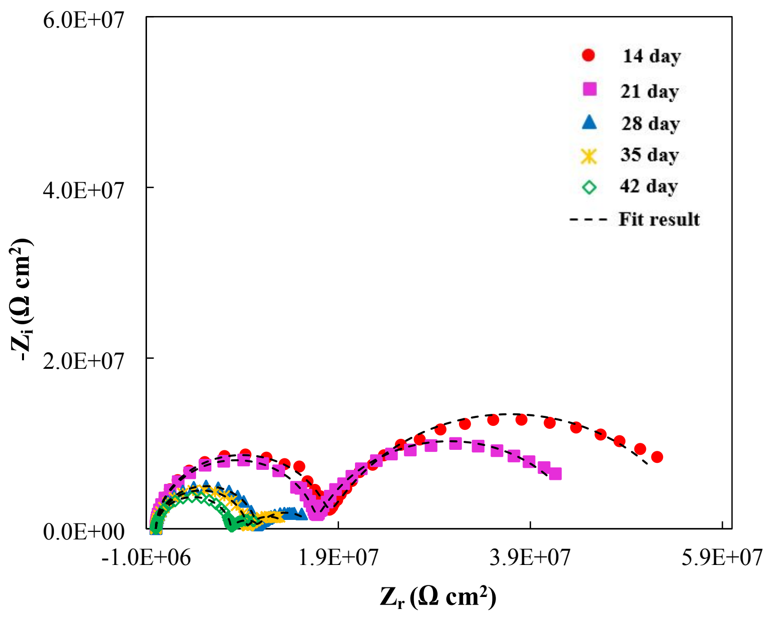

Figure S6. 2D Nyquist plots of the EP coatings following various immersion durations in 0.2 M HCl

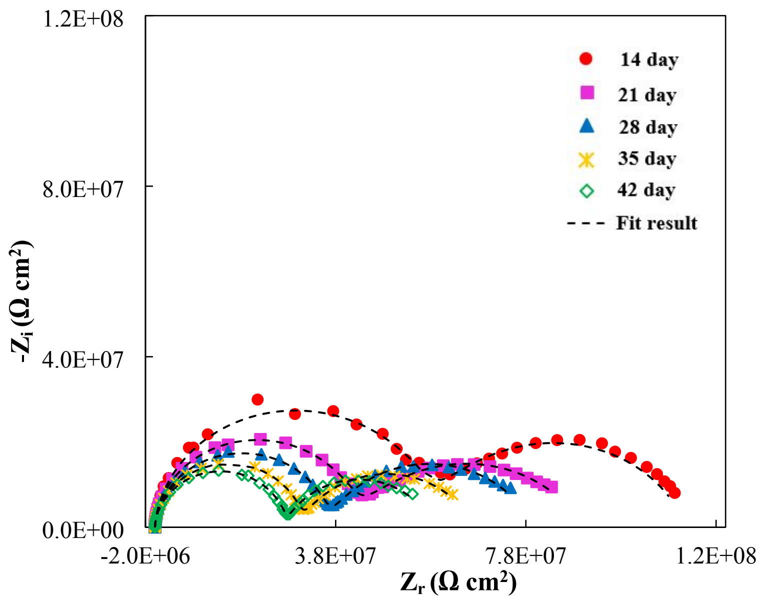

Figure S7. 2D Nyquist plots of the EP-MOF coatings following various immersion durations in 0.2 M HCl

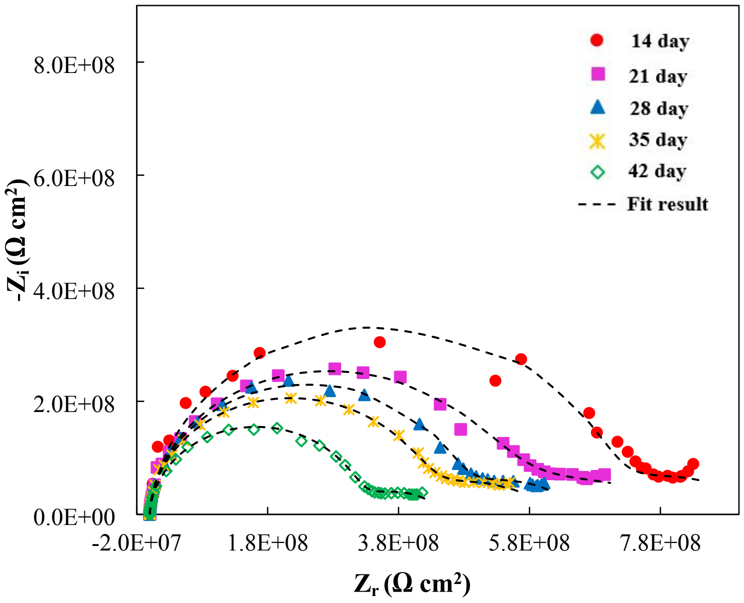

Figure S8. 2D Nyquist plots of the EP-EnMOF coatings following various immersion durations in 0.2 M HCl

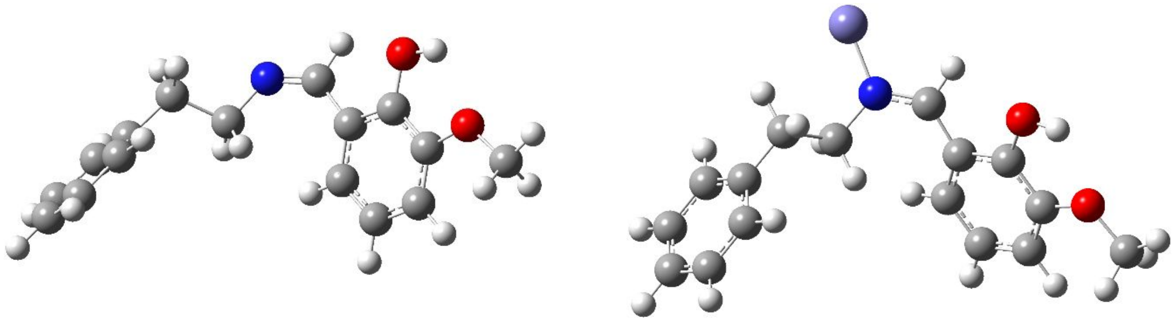

Figure S9. Optimized structures for pure Schiff base and Schiff base-Fe compounds in the solvated phase

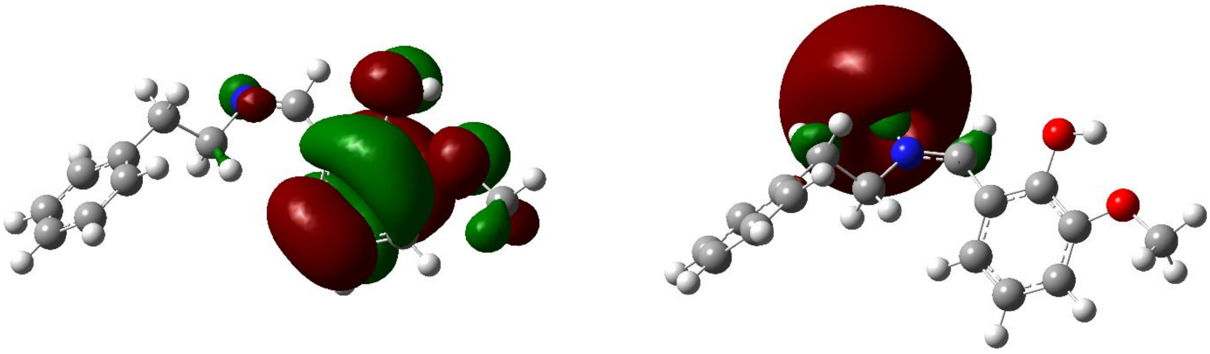

Figure S10. HOMO for pure Schiff base and Schiff base-Fe compounds in the solvated phase

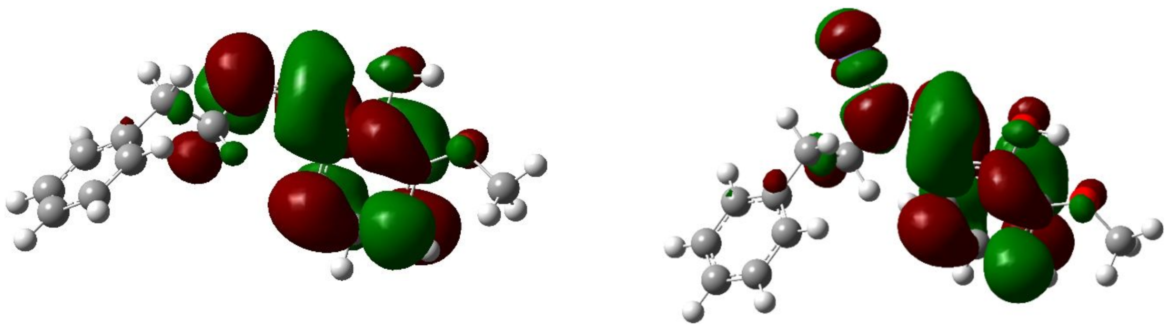

Figure S11. Optimized structures (a), HOMO (b), and LUMO (c) for pure Schiff base and Schiff base-Fe compounds in the solvated phase
